# Biofortification to avoid malnutrition in humans in a changing climate: Enhancing micronutrient bioavailability in seed, tuber, and storage roots

**DOI:** 10.3389/fpls.2023.1119148

**Published:** 2023-01-30

**Authors:** Sangam L. Dwivedi, Ana Luísa Garcia-Oliveira, Mahalingam Govindaraj, Rodomiro Ortiz

**Affiliations:** ^1^ Independent Researcher, Hyderabad, India; ^2^ International Maize and Wheat Research Center, Centro Internacional de Mejoramiento de Maíz. y Trigo (CIMMYT), Nairobi, Kenya; ^3^ Department of Molecular Biology, College of Biotechnology, CCS Haryana Agricultural University, Hissar, India; ^4^ HarvestPlus Program, Alliance of Bioversity International and the International Center for Tropical Agriculture (CIAT), Cali, Colombia; ^5^ Swedish University of Agricultural Sciences, Lomma, Sweden

**Keywords:** bioavailability, bioaccessibility and absorption, biofortified crop cultivars, climate change, genes and genetic markers, genetic engineering, nutrient acquisition, transport and storage

## Abstract

Malnutrition results in enormous socio-economic costs to the individual, their community, and the nation’s economy. The evidence suggests an overall negative impact of climate change on the agricultural productivity and nutritional quality of food crops. Producing more food with better nutritional quality, which is feasible, should be prioritized in crop improvement programs. Biofortification refers to developing micronutrient -dense cultivars through crossbreeding or genetic engineering. This review provides updates on nutrient acquisition, transport, and storage in plant organs; the cross-talk between macro- and micronutrients transport and signaling; nutrient profiling and spatial and temporal distribution; the putative and functionally characterized genes/single-nucleotide polymorphisms associated with Fe, Zn, and β-carotene; and global efforts to breed nutrient-dense crops and map adoption of such crops globally. This article also includes an overview on the bioavailability, bioaccessibility, and bioactivity of nutrients as well as the molecular basis of nutrient transport and absorption in human. Over 400 minerals (Fe, Zn) and provitamin A-rich cultivars have been released in the Global South. Approximately 4.6 million households currently cultivate Zn-rich rice and wheat, while ~3 million households in sub-Saharan Africa and Latin America benefit from Fe-rich beans, and 2.6 million people in sub-Saharan Africa and Brazil eat provitamin A-rich cassava. Furthermore, nutrient profiles can be improved through genetic engineering in an agronomically acceptable genetic background. The development of “Golden Rice” and provitamin A-rich dessert bananas and subsequent transfer of this trait into locally adapted cultivars are evident, with no significant change in nutritional profile, except for the trait incorporated. A greater understanding of nutrient transport and absorption may lead to the development of diet therapy for the betterment of human health.

## Global impact of malnutrition

**Figure 1:**
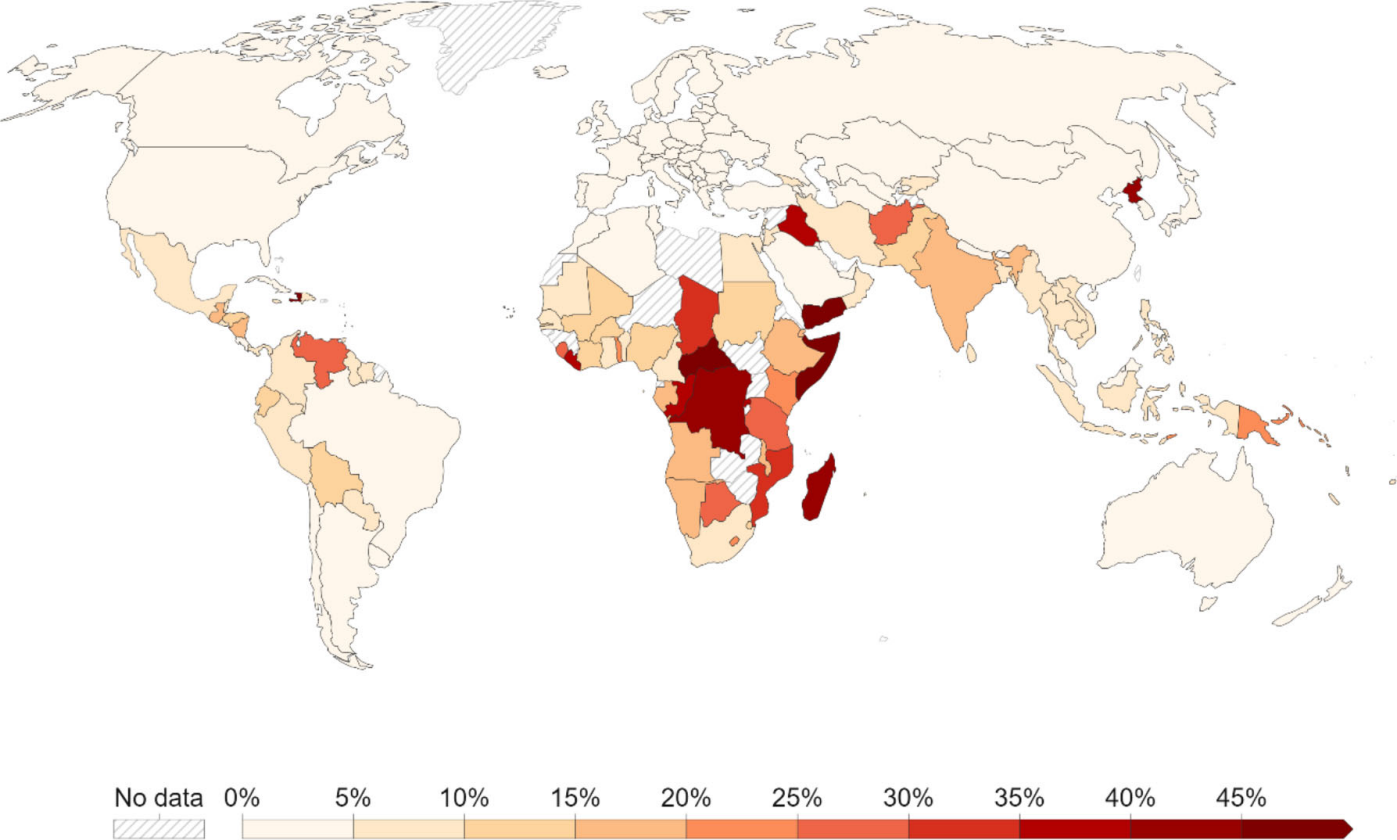
The prevalence of undernourishment (in percentage) in Global South (source: Asia, 2021; https://ourworldindata.org/hunger-and-undernourishment#long-term-decline-of-undernourishment).

The availability of essential vitamins and minerals is a fundamental requirement for human wellbeing, especially during the early stage of life. The lack of the right amount of vitamins, minerals, and other nutrients leads to the development of malnutrition. Malnutrition has different forms including wasting (low weight for height), stunting (low height for age), and underweight (low weight for age). All these ensue from poor-quality diets and may lead to non-communicable diseases. More than 2 billion people across the globe suffer from one or more micronutrient malnutrition (**Figure 1**; Von Grebmer et al., 2022). As per estimates from the World Health Organization (WHO) and the Food and Agriculture Organization (FAO) of the United Nations, 149 million children under 5 are stunted, 47 million are wasted, and 462 million are underweight. About 50% of child deaths under the age of 5 in developing countries are linked to undernutrition (FAO et al., 2020). Therefore, malnutrition affects physical and mental development, immunity, and overall health, thereby hampering human life potential largely in low- and middle-income countries (LMICs). The magnitude of micronutrient deficiency is particularly alarming among children, women of reproductive age, and pregnant and nursing mothers. Increased availability, accessibility, and affordability of dietary diversity including animal and dairy products address this complex food-system-based health problem. Food production is meant for both food and nutrition security. This has been ignored in recent agricultural research for development undertakings. The agricultural sector gained momentum in producing high yields through the Green Revolution efforts, but ignoring crop-based essential nutrition. Alternative agriculture should emphasize the production of food crops with better nutritional quality to minimize the risk of malnutrition in developing countries.

Given its impact on health, education, and economic productivity, persistent undernutrition is a major obstacle to human development, impacting the country’s prospects for future economic growth. To overcome this devastating situation of malnutrition, long-term efforts include fostering nutritionally dense cultivars that are highly prioritized alongside increasing yields and reducing negative consequences affecting yields in specific conditions. Globally, tackling the burden of malnutrition in all its forms remains challenging. For instance, the staggering impact of malnutrition in developing countries, in terms of gross domestic product (GDP), is almost 11% in Asia and Africa. The World Bank (2020) estimated that loss in GDP due to hidden hunger is up to 12 billion for India alone. The highest nutrition priority for eradicating malnutrition and achieving optimum nutrition for all is embedded in the National Nutrition Policy (NNP) adopted in 1993 by the Government of India, which acknowledges high prevalence of malnutrition according to a national family health survey (NFHS-V, 2021).

Improving nutrient levels in the edible parts of staple crops, by either crossbreeding, biotechnology, or agronomy, through the application of soil and leaf fertilizers, are cost-effective and sustainable approaches to reducing the burden of micronutrient deficiencies in the developing world (Garg et al., 2018). The CGIAR HarvestPlus Program has led nutrition research for development since 2003, by focusing on the top three nutrients with the highest deficiency among populations: iron (Fe), zinc (Zn), and vitamin A (HarvestPlus, 2022). Iron deficiency consequences are anemia, fatigue, weakness, and impaired cognition. Zinc deficiency leads to stunting in children, susceptibility to infections, and cell damage. Vitamin A deficiency contributes to impaired vision, night blindness, a higher risk of infection/death, and poor pregnancy outcomes. Nutrition is always given less priority in core crop improvement programs in national and international research and investments, except in a few time-bound bilateral projects. Thus, reducing the magnitude of malnutrition, especially in rural households, remains a big challenge. The prevalence of malnutrition after the COVID-19 pandemic is expected to significantly increase. The malnutrition prevalence levels are unacceptable in the Global South. The cost of addressing malnutrition is increasing in LMICs. An estimated 10 billion vitamin A capsules have been distributed to preschool children over two decades in LMICs (Tam et al., 2020). The importance of food security and improved nutrition is highlighted in the Sustainable Development Goals (SDG 2), which aim to end all forms of hunger by 2030 (Jose et al., 2020). The ongoing transition in CGIAR crop breeding and modernization investments highlight that improving nutrition traits is a priority to achieve SDG2.

## Global warming and elevated CO_2_ reduce the nutritional quality of food crops

Climate change and the quality of human diets are challenging issues and consequences for this century and upcoming ones. Increasing temperature and CO_2_ affects crop productivity, resilience, and nutritious quality. Semba et al. (2022) argued that climate change will affect micronutrient malnutrition by restricting the accessibility to micronutrient-rich crops. Furthermore, stoichiometric theory suggests that high CO_2_, as a rule, should alter the elemental plant composition (Loladze, 2002). The flour from cereals that were grown under elevated CO_2_ or using low nitrogen fertilizer amounts could lessen nutritional and processing quality and alter grain elemental composition (Erbs et al., 2010). It has also been noted that climate may reduce proteins (Jablonski et al., 2002; Stafford, 2007; Taub et al., 2008) and micronutrients in food crops (Stafford, 2007; Erbs et al., 2010; Scarpa et al., 2020). For example, as indicated by Hummel et al. (2018), elevated CO_2_ decreased iron intake by 4% for children below 5 years old and women of childbearing age, who eat common beans (*Phaseolus vulgaris* L.). Likewise, iron and zinc content in wheat (*Triticum aestivum* L.) grains were reduced by ca. 32% and 6%, respectively, under heat stress (Panigrahi et al., 2022). Loladze (2002) claimed that micronutrients may decline owing to “carbohydrate dilution” and reduce transpiration, thus affecting food quality and increasing micronutrient malnutrition, especially in the developing world.

Global warming lessens the uptake of micronutrients from the soil and the ensuing translocation within the plant (Maqbool et al., 2020). In this regard, high temperature negatively affects zinc acquisition in plants, thus resulting in poor nutritional quality. Ahmad et al. (2022) indicated that poor soil fertility management may further decrease the dietary supplementary supply of micronutrients. Likewise, owing to the ongoing COVID-19 pandemic, malnutrition has risen particularly in South Asia and sub-Saharan Africa (Sheoran et al., 2022). Hence, when it comes to biofortification, it must consider the target population of environments where the nutrient-dense bred germplasm will be further grown.

There are obvious interlinks between global warming, agri-food systems, and nutritional security (Bakker et al., 2021). Both agri-food systems and nutrition security are highly affected by climate variability and long-term climate change. The different types of foods consumed vary in their released greenhouse gases (GHGs), such as CO_2_ and NO_x_, and may promote change in land uses including an increase on clearing forests, draining wetlands, or tilling soil for agriculture, thereby contributing to climate change. This synergy steers different GHG emissions and distinct environmental footprints. A transdisciplinary approach should therefore be sought for developing an agri-food system that considers biofortification under rising temperatures, elevated CO_2_, and water stress, with the aim of ensuring food and nutrition security (Ebi et al., 2021). As shown by Huey et al. (2022), biofortification should be included in agri-food system interventions to overcome micronutrient deficiency in rural households. The microbiome may also provide a path towards nutrient-rich crops because of the known role of rhizospheres on crop nutrient dynamics (Tripathi et al., 2022).

## Physiological and molecular basis of nutrient acquisition, transport, and storage

Besides three basic non-mineral elements [carbon (C), hydrogen (H), and oxygen (O)], 14 mineral elements [nitrogen (N), phosphorus (P), potassium (K), calcium (Ca), sulfur (S), magnesium (Mg), chloride (Cl), iron (Fe), boron (B), manganese (Mn), zinc (Zn), copper (Cu), nickel (Ni), and molybdenum (Mo)] are broadly accepted as essential for the normal growth and development of plants. Based on the relative amounts needed for plant growth processes, these essential mineral elements can be classified into macro nutrients (primary: N, P, and K; secondary: Ca, S, and Mg) and micronutrients (Cl, Fe, B, Mn, Zn, Cu, Ni, and Mo), which can be found in plants at concentrations of >0.1% and <0.01% of dry tissue weight, respectively (Grusak et al., 2016). Being sessile, plants have not only evolved various physiological and molecular strategies for the uptake of essential micronutrients (trace minerals), such as iron and zinc from soil, but also developed tightly regulated mechanisms through coordination of several processes for their optimal distribution in between different organs as excess concentrations of these essential trace metals are toxic for their normal growth and development (Garcia-Oliveira et al., 2018).

Primarily, rhizosphere conditions have a decisive role in the adequate supply of mineral elements to meet the metabolic requirements of plants. Iron is one of the most abundant metals in soils but mainly present in the form of ferric (hydro)oxides, which have extremely low solubility in soil, resulting in restricted availability to plants. Plants uptake iron from rhizosphere in the form of either ferrous Fe(II), as in the case of non-graminaceous plants by acidification–reduction strategy (Strategy I), or ferric Fe(III) by graminaceous plants using chelation strategy (Strategy II), or a combination thereof (Marschner et al., 1986; Ishimaru et al., 2006). Most of the plants (dicot and non-graminaceous) including the model plant Arabidopsis (*Arabidopsis thaliana*) acidify the rhizosphere through the proton pump, H+-ATPase 2 (AHA2), and reduce ferric chelates Fe(III) to Fe(II) by the ferric reductase oxidase 2 (FRO2) enzyme (Robinson et al., 1999), and then, consequently, Fe(II) is taken up from soil through iron regulated transporter 1 (IRT1) (Eide et al., 1996). Graminaceous plants such as rice (*Oryza sativa* L.), maize (*Zea mays* L.), and wheat (*T. aestivum* L.) secrete mugineic acid (MA) family phytosiderophores (PS), which are produced from nicotianamine (NA) and chelate Fe(III), and subsequently, the Fe(III)–PS complex is transported into the root cells by Yellow Stripe 1 (YS1) or YS1-Like (YSL) transporters (Curie et al., 2001; Curie et al., 2009).

Like iron, total zinc content is also typical and relatively high in soil but occurs in various discrete chemical forms, and their solubility and availability to plants are influenced by the composition as well the physicochemical properties of the soil and biological factors, resulting, therefore, in reduced availability of zinc for plant absorption (White and Broadley, 2011). Zinc is acquired by roots from the soil solution into rhizodermal cells as free Zn^2+^ ion through members of the Zn-regulated transporter (ZRT) and IRT-like protein (ZIP) transporters. Soil zinc, like iron, is also solubilized *via* acidification and secretion of organic chelators (e.g., citrate, malate, and oxalate) or PS such as deoxymugineic acid (DMA) in the rhizosphere (White and Broadley, 2011). The precise uptake of Zn-chelate is not yet fully understood; nonetheless, the role of the YSL protein transporter has been demonstrated in the uptake of Zn-PS complexes in maize (von Wirén et al., 1996).

Following the initial uptake of iron and zinc from the soil solution, these are transported radially across the concentric root cell layers of epidermis, cortex, and endodermis. Once Fe reaches the central vasculature, it is then unloaded into the xylem where it is chelated with organic acids, especially citrate and MAs, and translocated to the shoots for long-distance transport (Durrett et al., 2007). For root-to-shoot transport, a multidrug and toxic compound extrusion (MATE) family transporter ferric chelate reductase defective 3 (FRD3) and ferroportin 1 (FPN1), also known as iron regulated 1 (IREG1) in Arabidopsis, respectively, load citrate and iron into the xylem (Durrett et al., 2007; Morrissey et al., 2009). Similarly, FRD-3 like 1 (FRDL1) in rice and Al-activated citrate efflux transporter 1 (AACT1) in barley, orthologs of Arabidopsis FRD3, and transporter of MAs (TOM) are crucial in graminaceous plants for iron transport in the xylem through the efflux of citrate and MAs, respectively (Furukawa et al., 2007; Yokosho et al., 2016). Similar to Fe, and for long-distance transport, it is also necessary to load Zn^2+^ ions into xylem vessels for its transport from root to the plant aerial parts. This task is known to be performed by heavy metal P1B-ATPase subfamily transporter (HMA; HMA2 and HMA4) proteins, and the members of ZIP and YSL families are thought to be involved in zinc loading into phloem (Chen et al., 2018a; Zlobin, 2021).

In the arial plant parts, YSL2 plays an important role in the lateral transport of Fe-NA complexes from the xylem to neighboring cells (DiDonato et al., 2004). Following xylem loading, Fe can be transferred to phloem for further long-distance transport and remobilization towards the crucial sink organs, especially leaves and seeds. Based on the analysis on the Fe complexes in the phloem sap, Nishiyama et al. (2012) suggested that DMA is the main Fe-chelating agent in the rice phloem sap, which indicates that switching of chelators occurs once Fe is loaded onto the phloem. In leaves, Fe re-enters the symplast and is reduced to Fe(II), mainly by the action of FRO proteins and used later for photosynthesis. In *Arabidopsis*, this process is mediated by an OligoPeptide Transporter 3 (OPT3) family protein and might play an important role in the transfer of both iron and zinc from vegetative organs to seeds (Kim and Guerinot, 2007).

During seed development, plants remobilize and move Fe/Zn from vegetative source organs into seeds. Iron is loaded into seeds as Fe(II) complexes by sequestration into vacuoles by vacuolar iron transporters (VIT1 in Arabidopsis and VIT1/VIT2 in rice) (Kim et al., 2006; Zhang et al., 2012) and into ferritin (Connorton et al., 2017a). The proportion of iron in vacuoles and ferritin differs according to plant species (Zielińska-Dawidziak, 2015). For instance, iron is mainly stored in vacuoles in the aleurone layer of cereal grains such as rice and wheat in the form of Fe-phytate complexes that are notoriously poorly bioavailable. Ferritin is predominantly located in the plastids and constitutes a highly bioavailable source of Fe; thus, high concentrations of ferritin in legume seeds make them a novel alternative dietary iron source (Zhao, 2010).

However, relatively little is understood about the transporters involved in zinc loading in seed. Yet, YSL1 and YSL3 have been implicated in phloem unloading of Zn in seeds of Arabidopsis (Waters et al., 2006; Chu et al., 2010). It is worth noting that the roles of HMA transporters (HMA2 and HMA4) are not exclusive to zinc loading into xylem vessels, but also might be responsible for the efflux of Zn from leaves (Stanton et al., 2022). Members of the cation diffusion facilitator (CDF) family, such as orthologs of metal tolerance proteins (MTPs) and Mg^2+^/H^+^ antiporter (MHX) in Arabidopsis, are thought to be involved in zinc loading in developing seeds by sequestering zinc into the vacuole, and subsequently regulates accumulation in the aleurone (Desbrosses-Fonrouge et al., 2005). Besides iron, VIT might be responsible in regulating zinc distribution between the flag leaf and seed tissues (Zhang et al., 2012; Connorton et al., 2017a). Among seed tissues, embryo and aleurone are major sources for zinc storage in cereal grains, but phytate present in these tissues is bound to zinc, resulting in the lower bioavailability of zinc.

Despite iron and/or zinc homeostasis in plants, model plants such as Arabidopsis and food grain crops have improved over the past decades (Kim and Guerinot, 2007; Connorton et al., 2017a; Garcia-Oliveira et al., 2018; Zlobin, 2021; Murgia et al., 2022; Stanton et al., 2022); such information is very limited in tuber and root storage crops. From a biofortification point of view, the genes whose functionality is linked to Fe/Zn metabolism and has been validated in various crops could be utilized in root and tuber crops by either exploring the novel alleles of such genes in the germplasm or employing directly transgenic approaches. For instance, the co-overexpressing *A. thaliana* iron transporter *IRT1* and ferritin *FER1* transgenic cassava lines, compared with controls (non-transgenic) under field conditions, showed up to 18 and 10 times higher iron and zinc content, respectively (Narayanan et al., 2019).

## Cross-talk between macro- and micronutrient transport and signaling in plants

In their normal life cycle, plants are frequently subjected to either deficiency or imbalance of nutrients, resulting in combined nutrient stresses. Various morphological, physiological, and agronomic studies clearly indicate the existence of mechanisms in plants that co-regulate these stresses. Nonetheless, metal nutrient cross-talk s at the molecular level have only been partly understood so far, and it will still take a long time to comprehensively understand the concept.

It is well established that interaction within or between macro- and micronutrients exists and can be grouped either as additive, antagonistic, or synergistic (Symeonidis and Karataglis, 1992). Antagonistic interactions between macro- and micronutrients (P and Fe, P and Zn) or within micronutrient (Fe and Zn, Fe and Mn, Zn and Cu) homeostasis have been reported at the physiological level. However, cross-talks between the signaling pathways integrating the homeostasis of these different essential mineral nutrients are still scarce.

At the molecular level, some mineral elements (divalent metals) compete for basic transporter proteins that have broad substrate specificity. For instance, IRT1 is the primary root iron transporter but also mediates the transport of Zn^2+^ and Mn^2+^, while NRAMPs support the transport of Fe^2+^ and Mn^2+^ (Rogers et al., 2000; Grotz and Guerinot, 2006). Similarly, ZIP and CDFs/MTP family proteins have also been reported to efflux Mn^2+^, Fe^2+^, and Zn^2+^ at the cellular level (Gustin et al., 2011). The transcription factor (leFER) that regulates the functions of Fe deficiency signaling was first identified in tomato (*Solanum lycopersicum*) plant (Ling et al., 2002). Subsequent studies identified FER-LIKE IRON DEFICIENCY INDUCED TRANSCRIPTION FACTOR (FIT) in Arabidopsis as the functional analog of FER, whose activity is regulated by a complex of the basic helix–loop–helix (bHLH) FIT, and at least one of the subgroup Ib bHLH proteins bHLH038, bHLH039, bHLH100, and bHLH101 (Gao and Dubos, 2021). FIT emerged as a central regulatory hub in root cells to steer and adjust the rate of Fe uptake and play an important role in fine-tuning Fe and Zn interactions (Chen et al., 2018b; Schwarz and Bauer, 2020). Recently, it has also been reported that FIT and bHLH Ib TFs, under iron deficiency conditions, do regulate the FRO4 and FRO5 genes involved in copper uptake (Cai et al., 2021).

From a better crop productivity perspective, P is considered one of the crucial macronutrients, but P in the form of Pi can continuously influence the bioavailability and mobility of Fe and Zn in soils (Briat et al., 2015; Xie et al., 2019). The transcription factor PHOSPHATE RESPONSE 1 (PHR1), originally found as a master regulator of P homeostasis in plants [which activates P deficiency response genes including transporters as well as regulatory RNA and proteins such as the micro-RNA miR399, IPS1, and the ubiquitin-conjugating E2 enzyme (PHO2)], also appeared as a regulator of the genes involved in the transport of both macronutrients such as S (sulfate transporters SULTR1;3, SULTR2;1, and SULTR3;4) and micronutrients including Fe (FER1 gene encoding the Fe storage protein ferritin) and Zn (ZIP2 and ZIP4 genes encoding zinc transporters) (Briat et al., 2015). Overall, the accumulation of omics data indicates that complex connections do exist between the various regulatory layers of the mineral nutrient homeostasis that balance the concentration of these essential micronutrients, at both the cellular and systemic levels.

## Nutrient profiling, spatial and temporal distribution, and accumulation

### Genetic variation

Plant genetic resources are the main source of variation from which researchers can draw functional allelic diversity for breeding cultivars showing a significant increase in nutrients that can bring health benefits. The greater emphasis in yield and uniformity led to the decline in crop diversity and the nutritional quality of food crops (Ortiz-Monasterio et al., 2007; Jacques and Jacques, 2012; Elgueta et al., 2021; Nicholson et al., 2021), whereas global warming may reduce the nutritional quality of food crops (Dwivedi et al., 2013; Scheelbeeka et al., 2018); Soares et al., 2019.

Substantial variation in cultivated gene pools for seed Fe and Zn concentrations in cereal and grain legume crops appears to be available (**Tables 1**, **2**), while root and plantain crops also show sufficient variability for β-carotene (provitamin A) contents (**Table 3**). Landraces were shown to contain greater amounts of seed Fe and Zn than modern cultivars. To date, many germplasm lines surpass the target values fixed by HarvestPlus (https://www.harvestplus.org). As an example, Russian spring bread wheat germplasm “Novosibirskaya 16”, “Silach”, “Line 4-10-16”, “Element 22”, and “Lutescens 248/01” had seed Zn content in the range of 46 –49 mg kg^−1^ seed dry weight (SDW) (Shamanin et al., 2021), while the provitamin A content of orange maize landrace from Malawi met the target level (15 μg g^−1^ SDW) of biofortification (Hwang et al., 2016). Several maize inbreds containing a high amount of β-carotene were also above the HarvestPlus biofortification target (Ashokkumar et al., 2020; Azmach et al., 2021). Yet, and despite these progresses, the environment (E) and genotype × environment (G ×E) interaction are still a major obstacle in breeding for improved seed quality traits, including seed Fe, Zn, and β-carotene (Khokhar et al., 2018; Kumar et al., 2019; Mengesha et al., 2019; Gangashetty et al., 2021; Gasura et al., 2021; Shamanin et al., 2021). A multilocation evaluation will help in identifying the locations that are favorable for the production of nutrient-dense food crops with specific quality attributes and identification of potential lines for varietal release.

**Table 1:** Genetic variation for seed iron (Fe) and zinc (Zn) contents among cereal crop germplasm.

Germplasm no.	Test environment no.	Micronutrient range variation	Reference
Barley			
216	2	Fe: 0.025 –0.047 mg g^−1^ seed; Zn: 0.029 –0.047 mg g^−1^ seed dry weight (SDW)	Thabet et al., 2022
496	2	Fe: 017– 0.043 mg g^−1^ SDW; Zn: 0.006– 0.071 mg g^−1^ SDW	Nyiraguhirwa et al., 2022
Maize			
1,813 inbreds	4	0.015– 0.036 mg g^−1^ SDW; Zn: 0.013– 0.052 mg g^−1^ SDW	Wu et al., 2021a
Pearl millet			
928 in 14 trials	14	Fe: 0.024 –0.145 mg g^−1^ SDW, Zn: 0.022 –0.096 mg g^−1^ SDW	Govindraj et al., 2022
281	2	Fe: 0.035– 0.116 mg g^−1^ SDW; Zn: 0.021– 0.080 mg g^−1^ SDW	Pujar et al., 2020
130	6	Fe: 0.032– 0.112 mg g^−1^ SDW; Zn: 0.027– 0.074mg g^−1^ SDW	Anuradha et al., 2017
Rice			
40	3	Fe (brown rice):0.005– 0.014 mg g^−1^ SDW; Zn (brown rice): 0.010– 0.029 mg g^−1^ SDW; Fe (polished rice): 0.001 –0.005 mg g^−1^ SWD; Zn (polished rice): 0.005– 0.023 mg g^−1^ SDW	Madhu Babu et al., 2020
192	2	Fe: 0.006– 0.023 mg g^−1^ and 0.001– 0.012 mg g^−1^, respectively, in brown rice (BR) and milled rice (MR): Zn: 0.011– 0.047 mg g^−1^ and 0.008– 0.041 mg g^−1^ in the BR and MR, respectively; maximum loss in grain Fe upon milling with a mean retention of 24.9% in milled rice, while Zn showed a greater mean retention of 74.2% in milled rice	Bollinedi et al., 2020
1,763	4	Fe: 0.001– 0.017 and 0.000– 0.026 mg g^−1^ SDW under flooded environments, respectively; Zn: 0.016– 0.065 and 0.019– 0.063 mg g^−1^ under unflooded environments, respectively	Pinson et al., 2015
126	–	Fe (brown rice): 0.006– 0.072 mg g^−1^ SDW; Zn (brown rice): 0.027– 0.067 mg g^−1^ SDW; with *O. nivara* and *O. rufipogon* having the highest Fe and Zn, respectively	Neelamraju et al., 2012
Sorghum			
242	2	Fe: 0.026– 0.049 mg g^−1^ SDW: Zn: 0.013– 0.043 mg g^−1^ SDW	Upadhyaya et al., 2016a
Wheat			
91	2	Fe: 0.028– 0.067 mg g^−1^; Zn: 0.029– 0.063 mg g^−1^; greater Fe and Zn in landraces than modern cultivars	Li-na et al., 2022
184	4	Fe: 0.025– 0.052 mg g^−1^ SDW; Zn: 0.017– 0.057 mg g^−1^ SDW	Rathan et al., 2022
166	4	Fe: 0.040 –0.055 mg g^−1^ SDW; Zn: 0.029– 0.051 mg g^−1^ SDW	Tong et al., 2022
49	6	Zn: 0.027– 0.030 mg g^−1^ SDW, with some sites showing greater Zn values than target value recommended by HarvestPlus	Shamanin et al., 2021
161	4	Fe: 0.042– 0.168 mg g^−1^ SDW; Zn: 0.030– 0.120 mg g^−1^ SDW	Liu et al., 2021b
245 Watkins collection	2	Zn: 0.024 –0.049 mg g^−1^ SDW; flours containing 0.008 –0.015 mg g^−1^ Zn and positively correlated with the wholegrain Zn concentration (*r* = 0.79) among 24 selected lines	Khokhar et al., 2020
31 M_7_ mutants	1	Fe: 0.041– 0.089 mg g^−1^ SDW; Zn: 0.022– 0.090 mg g^−1^ SDW; twofold higher Fe and Zn and 1.4- to 2-fold lower phytic acid compared to parent cv. Zhenis	Kenzhebayeva et al., 2019
330	6	Zn: 0.030–0.056 mg g^−1^ SDW, with greater variability at CIMMYT than low Zn in Indian environments	Velu et al., 2018
36	11	Zn: 0.025– 0.035 mg g^−1^ SDW	Khokhar et al., 2018
369	3	Zn: 0.025–0.053 mg g^−1^ SDW	Alomari et al., 2018
82	2	Fe: 0.041– 0.068 mg g^−1^; Zn: 0.036– 0.074 mg g^−1^; landraces having higher Zn than cultivars	Badakhshan et al., 2013

Original data converted into mg g^−1^ SDW.

**Table 2:** Genetic variation for seed iron (Fe) and zinc (Zn) contents among food legume crop germplasm.

Germplasm no.	Test environment no.	Micronutrient range variation	Reference
Chickpea			
92	4	Fe: 0.040– 0.091 mg g^−1^ SDW; Zn: 0.027– 0.062 mg g^−1^ SDW	Upadhyaya et al., 2016b
Common bean			
96	2	Fe: 0.067– 0.133 mg g^−1^ SDW; Zn: 0.021– 0.049 mg g^−1^ SDW	Nazir et al., 2022
1,512	4	Fe: 0.052– 0.115 mg g^−1^ SWD; Zn: 0.026– 0.047 mg g^−1^ SDW; best accessions exceeded the overall mean by 14-28%	Delfini et al., 2020
Groundnut			
64	8	Fe: 0.033– 0.068 mg g^−1^ SDW; Zn: 0.044– 0.095 mg g^−1^ SDW	Janila et al., 2014
184	3	Fe: 0.018– 0.031 mg g^−1^ SDW; Zn: 0.028– 0.044 mg g^−1^ SDW	Upadhyaya et al., 2012
Lentil			
96	3	Fe: 0.043– 0.111 mg g^−1^ SDW; Zn: 0.038– 0.082 mg g^−1^ SDW	Kumar et al., 2019
138	4	Fe: 0.053– 0.093 mg g^−1^ SDW; Zn: 0.028– 0.048 mg g^−1^ SDW	Khazaei et al., 2017
Pea			
299	Controlled environment	Fe: 0.031– 0.088 mg g^−1^ SDW; Zn: 0.022– 0.052 mg g^−1^ SDW	Powers et al., 2021
96	2	Fe: 0.047– 0.104 mg g^−1^; Zn: 0.027– 0.079 mg g^−1^	Hacisalihoglu et al., 2021

Original data converted into mg g^−1^ SDW.

**Table 3:** Genetic variation for β-carotene among maize, sorghum, and sweet potato germplasm.

Germplasm no.	Test environment no.	Nutrient range variation	Reference
Cereal crops			
Maize			
>3,000	2	Total carotenoids: −0.009– 0.854 mg g^−1^; β-carotene: −0.006 –0.055 mg g^−1^; β-cryptoxanthin: −0.006– 0.100 mg g^−1^; α-carotene: −0.007 –0.048 mg g^−1^ for untransformed BLUEs	Diepenbrock et al., 2021
24 (orange and yellow)	4	0.002– 0.015 mg g^−1^ SDW	Azmach et al., 2021
698 inbreds (10 sets)	–	0.012 – 1.78 mg g^−1^ SDW	Ashokkumar et al., 2020
26 landraces from Northeast India		0.000– 0.004 mg g^−1^ SDW among landraces from India’s Northeast Himalayan region; CAU-M66 and CAU-M16 with highest β-carotene, and well-adapted to stress environments	Natesan et al., 2020
		White and orange maize carotenoids averaged 0.002 and 0.059 mg g^−1^, respectively, with lutein most abundant (47.8%) in orange maize followed by zeaxanthin (24.2%), β-carotene (16.4%) and β-cryptoxanthin (11.6%); orange maize provitamin A met the target level (15 μg g^−1^ = 0.015 mg g^−1^) of biofortification	Hwang et al., 2016
129 (98 BC-derived lines + 7 recurrent parents +24 other adapted lines	3	β-carotene and provitamin A content among 25 best inbred lines ranged from 0.050 to 0.166 mg g^−1^ and from 0.080 to 0.174 mg g^−1^ SDW, respectively, with best BC-derived lines containing 23%–313% more β-carotene and 32%–190% more provitamin-A than the recurrent parents	Menkir et al., 2015
105 inbreds	Multilocation	Lutein 0.000– 0.011 mg g^−1^, zeaxanthin 0.000– 0.020 mg g^−1^ and β-carotene 0.000– 0.020 mg g^−1^, stable across locations	Muthusamy et al., 2015
		Substantial differences among the tropical-adapted yellow maize inbred lines for carotenoids, including provitamin A	Menkir et al., 2008
Sorghum			
447	1	β-carotene: 0.00003– 0.00119 mg g^−1^; lutein: 0.00002– 0.00461 mg g^−1^; zeaxanthin: 0.00001 –0.002 mg g^−1^	Cruet-Burgos et al., 2022
21 (yellow endosperm)	3	β-carotene: 0.0001– 0.0008 mg g^−1^; lutein: 0.0003 –0.0094 mg g^−1^; zeaxanthin: 0.0002–0.0091 mg g^−1^ SDW	Cruet-Burgos et al., 2020
100	?	Carotenoids: 0.0212– 0.0855 mg 100 g^−1^ SDW	Cardoso et al., 2015
Wheat			
247 (4 sets of germplasm)	–	0.096 –0.85 mg g^−1^ SDW	Ashokkumar et al., 2020
82	2	0.0009– 0.0017 mg g^−1^ SDW	Badakhshan et al., 2013
Root and plantain crops			
Cassava			
13 Nigerian landraces	2	β-carotene: 0.003– 0.361 mg g^−1^ root dry weight	Olayide et al., 2020
672	6	Linear relationship (0.81– 0.89) between yellow color intensity and carotenoid content; ~2/3 clones with white storage root types, the remainder with varying degrees of yellowness, suggesting distinct levels of carotenoid content; the average chromameter measure of yellow color intensity (b* value) was 20.8, ranging from 11.1 (white) to 40.8 (yellow)	Rabbi et al., 2017
23 (landraces)	1	Color of storage root varied from white to yellow to pink; total β-carotene ranged from 26.13% to 76.72%	Carvalho et al., 2016
Musa			
189	1	Total carotenoid ranged from 0.001 to 0.0362 mg g^−1^ fresh weight (FW), with 78% of carotenoids being β-carotene and α-carotene	Amah et al., 2019
48	1	Provitamin A: 0.0017– 0.0176 mg g^−1^ FW	Blomme et al., 2020
8	3	Total carotenoid: 0.0063– 0.037 mg g^−1^ fruit pulp (FP); β-carotene 0.0023– 0.0307 mg g^−1^ FP	Mbabazi et al., 2019
Sweet potato			
672	6	~2/3 clones with white storage roots, the remainder with varying levels of carotenoid content based on a range of yellow color; chromameter value (b* value), an indicator of yellow color intensity, ranged from 11.1 (white) to 40.8 (yellow)	Rabbi et al., 2017

Original data converted into mg g^−1^ SDW.

The development of mutant genetic resources, by either physical or chemical treatments, or targeted mutagenesis (TILLING, CASPR/Cas9 system), will allow the rapid detection of mutations in any gene of a genome (Holme et al., 2019). Knowing that the aleurone layer is the most nutritious part of cereal seeds, it is therefore desirable to seek targets to this seed portion to enhance the nutritional value of seeds. Concerning this, a rice mutant *thick aleurone 2-1* (*ta2-1*) with thickened aleurone [an average of 4.8 aleurone cell layers and up to 10 cell layers in some region of the seed in comparison to single-cell layer aleurone in the wild type (WT)] was shown to enhance the levels of multiple micronutrients simultaneously. Yet, the increases of aleurone-associated nutrients may not be proportional to the increases in the aleurone thickness; as minerals uptake, transport and loading capacity possibly affect their final content in aleurone (Liu et al., 2018), or larger sink (i.e., lower capacity in extra aleurone to synthesize or store nutrients compared to WT aleurone or the aleurone development) does not synchronize with the nutrient accumulation during seed development required for multiple micronutrients (Yu et al., 2021). Recently, a mutant line with a thickened aleurone layer, associated with higher rice bran nutritional value, was isolated in rice cultivar “Mizuhochikara” (Nguyen et al., 2022).

Genetically stable (M7) mutants in spring wheat broaden the genetic variation for seed nutrients, with Fe and Zn content ranging from 40.9 to 89.0 and 22.2 to 89.6 mg kg^−1^ SDW, respectively. The mutants also varied in Phy : Fe (1.40–5.32) and Phy : Zn (1.78–11.78) molar ratio. The higher the molar ratio, the greater the bioavailable levels of micronutrients (Kenzhebayeva et al., 2019). Targeting Induced Local Lesions in Genomes (TILLING) is a powerful strategy to introduce knockout mutations on *lcyE* gene (McCallum et al., 2000). Numerous knockout mutants *per* gene in protein coding regions and their sequence data are publicly available to unlock previously hidden variation in durum and bread wheats (Krasileva et al., 2017). TILLING-induced null mutant in wheat revealed strong reduction in the expression of *IcyE* but with pleiotropic effects on β-ring hydroxylase enzyme acting downstream in the pathway. Biochemical profiling relative to WT had ~75% increased β-carotene in the grains of wheat mutant line (Sestili et al., 2019). In durum wheat (*Triticum durum*), the combination of lycopene ε-cyclase and β-carotene hydroxylase 2 homoeologs significantly increased the β-carotene accumulation in mutant lines (Yu et al., 2022), thus proving mutational breeding as a powerful non-transgenic approach to enrich β-carotene in wheat seeds and possibly in other crops.

Although crop wild relatives were mainly exploited for host plant resistance to stress (Mammadov et al., 2018), a few wild relatives in barley, chickpea, rice, and sorghum were additionally identified as a rich source of seed Fe and Zn (Neelamraju et al., 2012; Abdelhalim et al., 2019; Wiegmann et al., 2019; Sharma S. et al., 2021). The examples include high seed Fe and Zn in *Oryza nivara* and *O. rufipogon* in rice (Neelamraju et al., 2012) and introgressed lines involving wild barley with more than 50% higher Fe and Zn content over barley cultivar, Barke (Wiegmann et al., 2019). Sorghum wild relatives, “Almahkara” and “Abusabiba”, respectively, had in the seeds the highest concentrations of total and bioavailable iron, 3.17 mg 100 g^−1^ and 92.8 mg 100 g^−1^, while another wild sorghum “Adar Umbatikh” grain had a higher zinc content (Abdelhalim et al., 2019).

Einkorn (*T. monococcum*) wheat, the wild emmer (*T. dicoccoides*), diploid progenitors of hexaploid wheat (*Aegilops tauschii*), *T. spelta*, *T. polonicum*, and wheat landraces are the most promising sources of seed Fe and Zn (Cakmak et al., 2004; Chhuneja et al., 2006; Sharma V. et al., 2021). Resynthesized hexaploid wheat originating from crosses between *T. durum* or *T. dicoccum* and diverse sources of *Ae. tauschii* offer a large pool of variability for agronomic as well nutritional traits (Velu et al., 2016), while a few derivatives such as “WB02” and “Zinc-Shakti” have 20% to 40% higher Zn than local controls (Singh and Velu, 2017). Several lines in the wheat genetic background of “Pavon 76”, originating from crosses involving rye (*Secale cereal*) translocation and *Ae Aegilops* species, are reported to contain greater Fe and Zn compared to controls (Velu et al., 2019). The wheat tetraploid (2n = 4× = 28 chromosomes) species *Triticum polonicum*, grown on a limited acreage in Spain, southern Italy, Algeria, Ethiopia, and warm regions of Asia, is characterized by the significantly highest seed Fe (39.1 mg kg^−1^) and Zn (49.5 mg kg^−1^) content (Bieńkowska et al., 2019).

In many countries, milled rice is the predominant consumed staple food. However, milling substantially reduces nutrients in the consumed grains, including Fe and Zn. Research previously done indicates multifold differences in Fe and Zn content between brown rice (BR) and milled rice (MR) among diverse germplasm (Bollinedi et al., 2020; Madhu Babu et al., 2020). Such germplasm is an ideal genetic resource to utilize toward developing Fe and Zn biofortified rice cultivars. The bioavailability of Fe is an issue that should be factored in while developing Fe biofortified cultivars. Yet, increasing Fe content in wheat seeds seems to not correlate with higher Fe bioavailability and the underlying genetic regions controlling Fe content not found, yet, to colocalize with increased Fe absorption. In addition, phytate content also does not correlate with Fe bioavailability, leading to the conclusion that phytate binding may be insufficient to explain the lack of correlation between Fe bioavailability and Fe content (Wright et al., 2021). All these observations give hope that Fe content and bioavailability are independent and feasible to integrate the two traits simultaneously. This opens doors for more research on exploring such variability in the germplasm pools to identify genotypes with high nutrient content and presenting higher levels of nutrient bioavailability.

### Diversity assessment

Assessing population structure and diversity among nutrient-dense germplasm offers researchers the opportunity to involve genetically diverse and nutritionally rich germplasm resources in crop breeding and genetics. A recent study involving advanced pearl millet breeding lines with threefold variation in panicle length, panicle girth, and 1,000-seed weight and large differences in seed Fe (35–116 mg kg^−1^ SDW) and Zn (21–80 mg kg^−1^ SDW) grouped 281 lines into 10 clusters, with cluster 5 showing the highest mean Fe, Zn, and 1,000-seed weight (Pujar et al., 2020). SNP-based diversity assessment differentiated 46 provitamin A maize inbred lines into two distinct clusters, with 0.60 average pairwise genetic distance and 0.359 average gene diversity (Kondwakwenda et al., 2020). The population structure of another set of 63 maize inbred lines bred for high levels of provitamin A revealed seven clusters with good agreement with Neighbor Joining clustering and was fairly correlated with pedigree and breeding origin (Sserumaga et al., 2019). The SSR-based diversity analysis of 24 maize inbred lines having wide variation in carotenoids, including β-carotene, containing *crtRB1* gene unfolded wide gene diversity (0.08 to 0.79) and dissimilarity coefficient (0.28 to 0.84) and grouped the inbreds into three distinct clusters (Duo et al., 2021). Phenotypic and genotypic diversity assessment of provitamin A cassava germplasm grouped 188 accessions into six and nine distinct clusters, respectively (Kamanda et al., 2020). The key message is genetically diverse nutrient-dense and agronomically superior inbred lines should be used to harness heterosis for seed nutritional traits when developing hybrids.

## Spatial and temporal distribution and accumulation of Fe and Zn

A large part of the population in less favored parts of the world is dependent on the consumption of grain-based food products for their calorie and nutrient need. Understanding the spatial distribution and localization of minerals within the edible parts of plant products may contribute to their nutritional improvement. A genetically biofortified wheat had a higher concentration of Fe and Zn in the seed. The location of the minerals is similar in both biofortified and non-biofortified wheat. Fe is abundant in the aleurone layer, while Zn is highly concentrated in the embryo. Developing seeds show a decreasing trend in concentration from the proximal to the distal ends. Phytic acid, present in these tissues, binds minerals as phytate. Minerals bound to phytate have poor bioavailability (Wan et al., 2022). Fe and Zn in maize primarily accumulate in the scutellum of the embryo during early seed development, with trace amounts detected in the aleurone layer at mature seed stage. P also accumulated in the scutellum, but no specific pattern was detected with Fe and Zn accumulation; Fe and Zn accumulated linearly in maize scutellum (Cheah et al., 2019).

Assessing the spatial patterns of Fe and Zn accumulation during grain development in barley lines with contrasting Zn concentrations revealed a gradual decrease in Zn concentrations from the aleurone to the endosperm, while Fe and P decreased sharply. Fe colocalized with P in the aleurone, and Zn with sulfur in the sub-aleurone. Thus, endosperm storage capacity largely influences differences in seed Zn concentrations (Detterbeck et al., 2020). A study of spatial distribution of minerals between and within the maternal and filial tissues in diverse wheat genotypes (cultivar, landrace, and wild species) revealed aleurone and scutellum as the major storage tissues for macro- and micronutrients. The genotypes showed distinct elemental distribution patterns, grouped into four distinct clusters. Wild relative (*A. kotschyi*) and landrace (IITR26) accumulated more Zn and Fe in scutellum and aleurone than wheat cultivars (WH291 and WL711). Other nutrients also showed a distinct distribution pattern (Singh et al., 2014).

In an experiment involving high yielding wheat cultivars but with contrasting seed Zn concentrations, Guo et al. (2021) noted 103%, 76%, 64%, 50%, and 33% higher Zn concentrations in the crease region, scutellum, endosperm, aleurone layer, and embryonic axis, respectively. Zn colocalized with P in the aleurone layer and the scutellum, but less colocalization of Zn with P and a much lower concentration of P:Zn ratio in the high-Zn cultivar. Thus, a lower proportion of P:Zn ratio in the high-Zn cultivar suggests the feasibility of combining high grain yield with high Zn content and high bioavailability.

Iron trafficking in plants is key to improving the nutritional quality of food crops. Sheraz et al. (2021) used iron-57 (^57^Fe) isotope labeling and NanoSIMS to visualize iron translocation between tissues and within cells in immature wheat seed between a *TaVIT2* overexpressing line and WT. Fe transports from maternal tissues to the embryo through the different cell types and storage in vacuoles, with most of the Fe detected in intracellular bodies, indicating symplastic rather than apoplastic transport. The presence of highly enriched ^57^Fe in aleurone cells suggests iron being delivered to phytate globoids. This endosperm-specific expression of *TaVIT2* may have relevance to developing new biofortification strategies in cereal crops.

How does Fe accumulate in embryo in *Brassica* species seeds? Perls/DAB staining is effective to localize iron at the cellular and subcellular levels. *Brassica* seed species, except for *Vasconcellea pubescens*, which accumulate and store Fe in cortex cell, accumulate Fe in nuclei in specific stages of embryo maturation before it is localized in vacuoles of cells surrounding provasculature in mature seeds, specific to *Brassicales*, not widely found in other Eudicotyledoneae (Ibeas et al., 2017; Ibeas et al., 2018). Surely, understanding where and how Fe and Zn accumulate in seed crops may assist in the development of biofortified staple crops with increased bioavailability of micronutrients.

### Trait inheritance

#### Functionally characterized genes associated with Fe and Zn

Functionally characterized genes are those whose genetic functions have been determined either through transgenesis, RNAi approach, mutagenesis, or targeted gene editing (Alberts et al., 2002; Agrawal et al., 2003; Kamburova et al., 2017).

Cereal endosperm is a major source of dietary energy. It is well known that milling substantially reduces the nutritional quality of milled seeds or white flour. Enhancing the nutritional value of endosperm though biotechnological interventions may provide nutrient-dense seed or flour. Many functionally characterized genes are associated with seed Fe and Zn in cereals (**Table 4**). For example, barley transgenic lines containing *HvMTP1* under the control of endosperm-specific promoter showed significant enhancement of Zn in the endosperm (Menguer et al., 2018). Transgenic rice expressing three (*AtNRAMP3*, *AtNAS1*, and *PvFER*) or two (*AtNRAMP3* and *PvFER*) genes under the control of the endosperm-specific promoter increased Fe and Zn close to the recommended levels, with a maximum in milled grains with three genes (Wu et al., 2019). Wheat plants expressing *OsNAS2* accumulated greater concentrations of Fe and Zn in endosperm and crease tissues (Beasley et al., 2019), while those expressing *TaVIT2* enhanced Fe more than twofold in white flour fraction without an increase in phytate, with more bioavailable Fe (Connorton et al., 2017b).

**Table 4:** Functionally characterized genes associated with increased seed Fe and Zn contents in cereal and food legume crops.

Gene	Grain (or white flour) Fe and Zn contents	Reference
Cereals		
*ZmIRT2*	Transgenic maize overexpressing *ZmIRT2* elevated Zn and Fe levels in roots, shoots, and seeds; *ZmIRT1, ZmIRT2*, and *ZmYS1* function cooperatively to maintain Zn and Fe homeostasis in *ZmIRT2* overexpressing plants	Li et al., 2022
*TaVIT2-D*, *OsNAS2*	A wheat line containing *VIT2-NAS2* construct led to a twofold increase in Zn to ∼50 µg g^−1^ in wholemeal flour and a twofold increase in both Zn and Fe in hand-milled white flour, while Fe increased by threefold to ∼25 µg g^−1^ in roller-milled white flour; indistinguishable growth between of VIT-NAS and WT plants in the greenhouse	Harrington et al., 2023
*OsNAS2*	Constitutive expression of *OsNAS2* upregulated biosynthesis of NA and DMA involved in metal transport; *OsNAS2* plants accumulated higher concentrations of Fe and Zn in endosperm and crease tissues of wheat grain; Fe bioavailability enhanced in white flour	Beasley et al., 2019
*AtNRAMP3*, *AtNAS1*, *PvFER*	Transgenic rice expressing *AtNRAMP3* together with *AtNAS1* and *PvFER*, or expressing only *AtNRAMP3* and *PvFER* together increased Fe and Zn close to recommended levels; maximum when *AtNRAMP3*, *AtNAS1*, and *PvFER* expressed together (Fe: 12.67 μg g^−1^ SDW; Zn: 45.60 μg g^−1^ SDW) in polished grains, >90% of the recommended iron increase in endosperm	Wu et al., 2019
*OsHMA7*	Overexpression of *OsHMA7_261_* allele increased grain Fe and Zn but with significant yield penalty; overexpression of *OsHMA7*_284_ allele resulted in lines with either high-grain Fe and Zn contents and tolerant to Fe and Zn deficiency or low-grain Fe and Zn contents and sensitive to Fe and Zn deficiency; knocking down expression of *OsHMA7* by RNAi silencing resulted in lines with altered domestication traits and increased sensitivity to Fe and Zn deficiency	Kappara et al., 2018
*HvMTP1*	Barley transgenic lines containing *HvMTP1* under the control of the endosperm‐specific D‐hordein promoter were no different in growth but had higher grain Zn compared to WT; significant enhancement of Zn in the endosperm provides opportunities to enrich cereals endosperm with Zn	Menguer et al., 2018
*OsNAS2, PvFERRITIN*	*OsNAS2* and *PvFERRITIN* expression, as single genes or in combination, resulted in a significant increase of Fe and Zn contents in wheat grains; lines expressing *OsNAS2* substantially surpass the HarvestPlus recommended target [30% dietary estimated average requirement (EAR) for Fe and 40% of EAR for Zn], with lines containing 93.1 and 140. 6 µg g^−1^, respectively	Singh et al., 2017
*TaVIT2*	Overexpressing wheat plant containing *TaVIT2* under the control of an endosperm-specific promoter achieved more than twofold increased Fe in white flour fractions (endosperm fraction) with no increase in phytate, and bioavailable Fe in white flour fraction	Connorton et al., 2017b

#### Quantitative trait loci and candidate genes associated with seed Fe and Zn

Advances in next-generation sequencing including genotype-by-sequencing (GBS) and development on bioinformatics resources to process large data sets, both genotyping and phenotyping, to store, retrieve, analyze, interpret, and share have facilitated researchers to identify abundant SNP-based markers, assess marker–trait associations (MTAs) through genome-wide association studies, develop high-density genetic maps, and fine-map to clone and functionally characterize genes. Diversity germplasm panels and advanced generations’ recombinant inbred lines (RILs) including those from multi-parent advanced generation inter-cross (MAGIC) and nested association mapping (NAM) panels in most crops serve as enduring resources to genetic analysis of complex traits (Perea et al., 2016; Arrones et al., 2020; Jan van Dijk et al., 2021; Wang et al., 2021; Zhao et al., 2021).

Numerous SNP-based significant MTAs, quantitative trait loci (QTLs), and candidate genes associated with seed Fe and Zn content are reported in cereal and legume crops, and a few QTL with major effects (**Tables 5**, **6**). SNP was physically located within genes involved in seed Fe and Zn homeostasis (Thabet et al., 2022) or SNP in close vicinity to two yellow stripe-like (YSL) genes was associated with grain Zn (Detterbeck et al., 2019) in barley. QTL hotspots simultaneously affected multiple grain nutrients, and an exotic allele linked to *HvGA20ox_2_* significantly increased multiple grain elements with no yield penalty in maize (Herzig et al., 2019), while in pearl millet, candidate genes related to Fe and Zn metabolism correlated with known QTL regions for grain Fe and Zn contents (Mahendrakar et al., 2020). *qGZn9a* linked to high-grain Zn in *Oryza meridionalis* also adversely affects fertility levels. One of the eight candidate genes in *qGZn9a* specifically expressed in the developing anther and possibly regulated anther dehiscence, which suggests that a balancing selection is needed to ensure simultaneous improvement in rice (Ogasawara et al., 2021), while haplotype-based association mapping involving 65 genes related to Zn responses on diverse germplasm panels and MAGIC populations revealed diversity panel- or population- specific candidate genes associated with high seed Zn content (Liu et al., 2021a). Many of the candidate genes associated with Fe and Zn uptake, transport, storage, and regulation in wheat were orthologs of known *Arabidopsis* and rice genes related to Fe and Zn homeostasis (Tong et al., 2022), while SNPs mapped on chromosome 1A and 2A in wheat genome significantly associated with seed Fe and Zn content had no adverse effects on seed weight (Liu et al., 2021b). *CaqFe4.4*, *CaqFe4.5*, and *CaqZn4.1* associated with seed Fe and Zn collocated in the “QTL-hotspot” region on CaLG04 harboring several drought tolerance QTL, adding advantage to effect selection for greater Fe and Zn content combined with drought tolerance in chickpea (Sab et al., 2020). A critical follow-up step would be the functional validation of such putative candidate genes prior to these genes being directly deployed in crop improvement programs.

**Table 5:** Candidate genes/QTLs and marker–trait association (MTA) SNPs associated with increased grain Fe and Zn contents in cereal crops.

Candidate genes/QTLs and marker–trait association	Reference
Cereals	
Barley	
High LD-SNPs with Fe and Zn, strongest SNP physically located within genes involved in grain Zn and Fe homeostasis; BHLH family TF and Squamosa promoter binding-like protein as potential candidate genes	Thabet et al., 2022
Twelve and 6 SNPs associated with grain Zn and Fe, respectively	Nyiraguhirwa et al., 2022
Thirteen shared MTA, with significant MTA on 2H in close vicinity to two yellow stripe-like (YSL) genes associated with grain Zn	Detterbeck et al., 2019
Maize	
*nas5*, involved in the synthesis of a metal chelator, nicotianamine, associated with both Fe and Zn	Wu et al., 2021a
QTL hotspots simultaneously affect multiple grain nutrients; exotic allele linked to *HvGA20ox_2_* significantly increased multiple grain elements with no yield penalty	Herzig et al., 2019
Pearl millet	
Fourteen QTLs for grain Fe and 8 for Zn across environments; Ferritin gene, Al^3+^ Transporter, K^+^ Transporters, Zn^2+^ transporters, and Mg^2+^ transporters as candidate genes	Singhal et al., 2021
29 candidate genes related to Fe and Zn metabolism correlated with known QTL regions for grain Fe/Zn; tissue and stage-specific expressions; Fe and Zn: *PglZIP*, *PglNRAMP*, and *PglFER*	Mahendrakar et al., 2020
Rice	
*qGZn9a* linked with high-grain Zn levels in *O. meridionalis* W1627 but with reduced fertility levels; a partial defect in anther dehiscence correlates with increased grain Zn; eight candidate genes in the *qGZn9a* region, with one specifically expressed in the developing anther and possibly regulates anther dehiscence; thereby, a balancing selection is needed to effect simultaneous improvement	Ogasawara et al., 2021
Two QTL *qZPR.1.1* and QTL *qZPR.11.1* on chromosome 1 and a common QTL on chromosome 2 for grain Zn in polished rice; two candidate genes related to transporters	Suman et al., 2021
Haplotype-based association mapping involving 65 genes related to Zn responses on three diverse panels revealed five (*OsNRAMP6*, *OsYSL15*, *OsIRT1*, *OsIDEF1*, and OsZIFL7; PVE 7.70%–15.39%), three (*OsFRDL1*, *OsIRT1*, and *OsZIP7*, 11.87%–17.99%), and two (*OsYSL7* and *OsZIP7*, 9.85%–10.57%) genes, respectively, associated with grain Zn in diversity panels from Southeast Asia (SEA) and South China (SC), and MAGIC population; haplotype analysis revealed that *Hap1-OsNRAMP5*, *Hap5-OsZIP4*, *Hap1-OsIRT1*, *Hap3-OsNRAMP6*, *Hap6-OsMTP1*, and *Hap6-OsYSL15* had the largest effects for Zn in SEA and *Hap3-OsOPT7*, *Hap4-OsIRT2*, *Hap4-OsZIP7*, *Hap5-OsIRT1*, and *Hap5-OsSAMS1* had the most significant effects in the SC panels	Liu et al., 2021a
Zn in polished grains associated with SNPs located in three putative candidate genes on chromosomes 3 and 7; chromosome 7 genomic region colocalized with previously identified genomic regions (rMQTL_7_._1_) and OsNAS3 candidate gene	Babu et al., 2020
Six, 7, 11, and 5 significant MTAs with grain Fe in brown rice (BR), milled rice (MR), and grain Zn in BR and MR, respectively; PVE: 2.1%– 53.3%	Bollinedi et al., 2020
Swarna × *O. nivara* RILs: 5 and 3 QTLs, respectively, for Fe and Zn each contributed >15% phenotypic variance (PV); candidate genes *OsYSL1*, *OsNAC*, *OsYSL16*, *OsZIP4*, *OsYSL17*, and *OsNAAT1* underlie the Fe or Zn QTLs; Madhukar × Swarna RILs: 7 and 6 QTLs, respectively, for Fe and Zn explaining >30% PV; candidate genes *OsYSL1*, *OsNAC*, *OsYSL16*, *OsZIP4*, *OsYSL17*, and *OsNAAT1* underlie the Fe or Zn QTLs	Neelamraju et al., 2012
*O. rufipogon* × Teqing 85 ILs: two grain Fe (5–7) and three grain Zn (5–19) QTLs for Fe and Zn contributed with 5%–7% and 5%–19% of PV, respectively; colocalized with other nutrient QTLs, including Ca and Mg.	Garcia-Oliveira et al., 2009
Sorghum	
Three QTLs (*qfe7.1*, *qzn7.1*, and *qzn7.2*) mapped on SBI-07 across environments for grain Fe and Zn; two putative candidate genes, CYP71B34 and ZFP 8	Kotla et al., 2019
Wheat	
Four and two significant MTAs, respectively, associated with grain Fe and Zn; F-box-like domain superfamily, Zinc finger CCCH-type proteins, serine-threonine/tyrosine-protein kinase, histone deacetylase domain superfamily, and SANT/Myb domain superfamily proteins as candidate genes underlying identified genomic regions	Rathan et al., 2022
Eight candidate genes for Zn/Fe uptake, 11 for transport, 3 for storage, and 6 for regulations; 11 of these putative wheat orthologs of known *Arabidopsis* and rice genes related to Zn/Fe homeostasis	Tong et al., 2022
Three and five MTAs on 1A and 2A significantly associated with grain Zn and Fe contents, with no adverse effects on grain weight; 38 candidate genes into four classes: enzymes, transporter proteins, MYB transcription factor, and plant defense responses proteins	Liu et al., 2021b
Seven pleiotropic QTL for grain Zn and Fe on 1B, 1D, 2B, 6A, and 7D; several candidate genes underlying QTL, including those to the families of the transporters and kinases known to transport small peptides and minerals and catalyzing phosphorylation processes	Rathan et al., 2021
Thirty-nine MTAs for grain Zn, with two larger effect QTL regions on chromosomes 2 and 7; zinc finger motif of transcription factors and metal-ion binding genes associated with QTL	Velu et al., 2018
A total of 161 significant and consistent MTAs located on chromosomes 3B and 5A with major effects; putative candidate genes related to Zn uptake and transport or represent bZIP and mitogen-activated protein kinase genes	Alomari et al., 2018

**Table 6:** Candidate genes/QTLs and marker–trait association (MTA) SNPs associated with increased seed Fe and Zn contents in food legume crops.

Candidate genes/QTLs and MTA	Reference
Common bean	
A total of 113 SNPs associated with seven micronutrients including Fe and Zn (13.50%– 21.74% PVE) mapped on chr3 and chr11	Nazir et al., 2022
Chickpea	
Three QTLs for seed Fe and Zn contents (*CaqFe4.4, CaqFe4.5*, and *CaqZn4.1*) colocated in the “*QTL-hotspot*” region on CaLG04 harboring several drought tolerance QTLs; candidate genes encoding iron–sulfur metabolism and zinc-dependent alcohol dehydrogenase activity on CaLG03, iron ion binding oxidoreductase on CaLG04, and zinc-induced facilitator-like protein and ZIP zinc/iron transport family protein on CaLG05	Sab et al., 2020
Sixteen genomic/gene loci associated (29% combined PVE) with seed-Fe and Zn concentration, with 11 trait-associated SNPs linked tightly with eight QTLs	Upadhyaya et al., 2016b
Lentil	
Two and one SNP tightly linked to seed Fe and Zn concentrations, respectively	Khazaei et al., 2017
Pea	
Multiple significant SNPs and candidate genes associated with grain Fe and Zn	Powers et al., 2021
Soybean	
Five QTLs each for seed Fe (4.57%– 32.71% PVE) and Zn (3.35%– 26.48%) contents; 12 candidate genes in one major QTL for seed Fe content; two major QTLs for seed Zn content	Wang et al., 2022

## Carotenoid biosynthesis in plants

Carotenoids are synthesized in plant plastids and undergo a series of enzymatic reactions to produce carotenoids with varying color and composition that accumulate in a specialized organelle, the amyloplast, the major site for starch synthesis and storage in seed endosperm (Sun et al., 2018). Provitamin A, the intermediate product of biosynthetic pathway characterized by un-hydroxylated β-rings, is converted to retinol (or vitamin A) in the body *via* oxidative cleavage and storage in the lever (Stahl and Sies, 2005). Two retinyl groups of “β-carotene” and one retinyl group of “β-cryptoxanthin” and “α-carotene” are the most abundant provitamin A carotenoids in plant-based foods (Cazzonelli and Pogson, 2010). Carotenoid biosynthesis pathways and genes are highly conserved across plants, including the model plant *Arabidopsis* (Ruiz-Sola and Rodríguez-Concepción, 2012; Owens et al., 2014).

Phenotypic diversity for carotenoid profiles is extensively characterized in maize (**Table 3**). Yellow- and orange-colored seeds are the source of carotenoids. The first step in carotenoid biosynthesis is the five-carbon central intermediate isopentenyl pyrophosphate (IPP) produced by methyl-D-erythritol-4-phosphate (MEP) in the plastid. The condensation of two 20-carbon geranylgeranyl diphosphate (GDDP) molecules by the phytase synthase enzyme forms phytoene. Phytoene is sequentially desaturated and isomerized to produce lycopene. Lycopene is cyclized with two β-rings to form β-carotene or with one β-ring and one α-ring to form α-carotene. α-carotenes and β-carotenes are hydroxylated twice to form, respectively, lutein and zeaxanthin. Zeaxanthin is then modified to yield violaxanthin and neoxanthin (Diepenbrock et al., 2021). Eleven out of 44 QTLs were resolved to individual genes (*vp5*, *lut1*, *im1*, *zep1*, *psy1*, *dxs2*, *lcyE*, *dxs3*, *ccd1*, *crtRB5*, and *crtRB1*) regulating IPP biosynthesis and carotenoid biosynthesis and degradation. Six of these were correlated expression and effect QTLs (ceeQTLs), with strong correlation between RNA-seq expression abundances and QTL allelic effect estimates across six stages of seed development and largest PVE (%). Three to five loci captured the bulk of genetic variation for each trait. Most of these ceeQTLs had strongly correlated QTL allelic effect estimates across multiple carotenoid traits. A few genes (*vp5*, *im1*, *dxs2*, and *dxs3*) in the IP pathway and early steps of the carotenoid pathway that reside upstream of lycopene cyclization, not previously associated with natural variation in maize seed carotenoids (Harjes et al., 2008; Azmach et al., 2018; Baseggio et al., 2020; Diepenbrock et al., 2021), are the potential new targets in maize breeding. Thus, an in-depth genome-level understanding of the genetic and molecular control of carotenoids may provide a roadmap to accelerate breeding for provitamin A and other carotenoids in maize seed (Diepenbrock et al., 2021). An earlier study reported eQTLs for 4 of the 11 genes (*lcyE*, *crtRB1*, *ccd1*, and *vp5*), 2 of which (lcyE and crtRB1) are represented in the six ceeQTLs (Diepenbrock et al., 2021), and *lcyE* and *crtRB1* expression significantly correlated with provitamin A concentrations (Fu et al., 2013).

## Global efforts to breed nutrient-dense crops

### Cross breeding and genomic-assisted breeding

Biofortification has been pursued actively in crop improvement programs of the Global South (Asia, 2021; **Figure 1**) in the last two decades. To demonstrate that biofortification breeding is successful in product delivery through regular testing and release networks, a strong breeding strategy was established by CGIAR HarvestPlus based on three stage-gate tasks such as discovery, development, and dissemination (Bouis et al., 2011; Saltzman et al., 2014; Bouis and Saltzman, 2017; Pfeiffer et al., 2018; Govindaraj et al., 2019).

Discovery is an initial stage to assess the biofortification breeding potential of each crop. The essential component includes identification of the target population and staple food (crop) consumption, establishing a breeding baseline and nutrient target levels, and screening and characterizing germplasm for target nutrients. The outcomes of the discovery phase will guide the product development stage for target nutrients in each crop. The essential stage is for developing biofortified cultivars with target levels. This development stage includes breeding pipelines, managing the genotype × environment (G × E) interaction on target nutrients, nutrition bioavailability assessment, and efficacy research involving human feeding trials. The delivery stage is for product release and assessing a farmer’s field performance and consumer preferences. The investment made in biofortification trait breeding and product development will be realized based on the final product release and incremental increase in the target minerals or vitamins over the regular or popular cultivars. Hence, key components are the release of biofortified products, dissemination, and market and consumer acceptance. The impact of biofortified crops can be further assessed by measuring the improved nutritional status of the target population after regular human consumption of their derived food.

**Figure 2:**
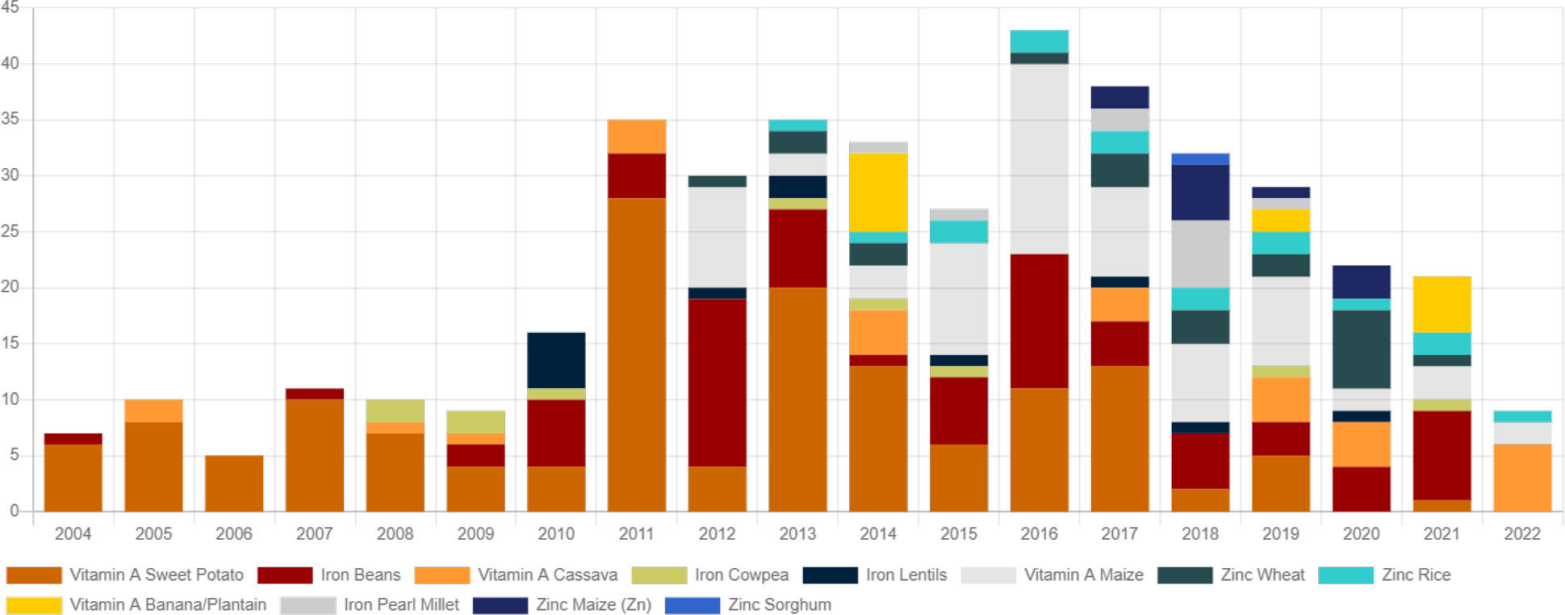
Nutrient-rich bred cultivars released in different crops for the last two decades (source: CGIAR HarvestPlus).

**Figure 3:**
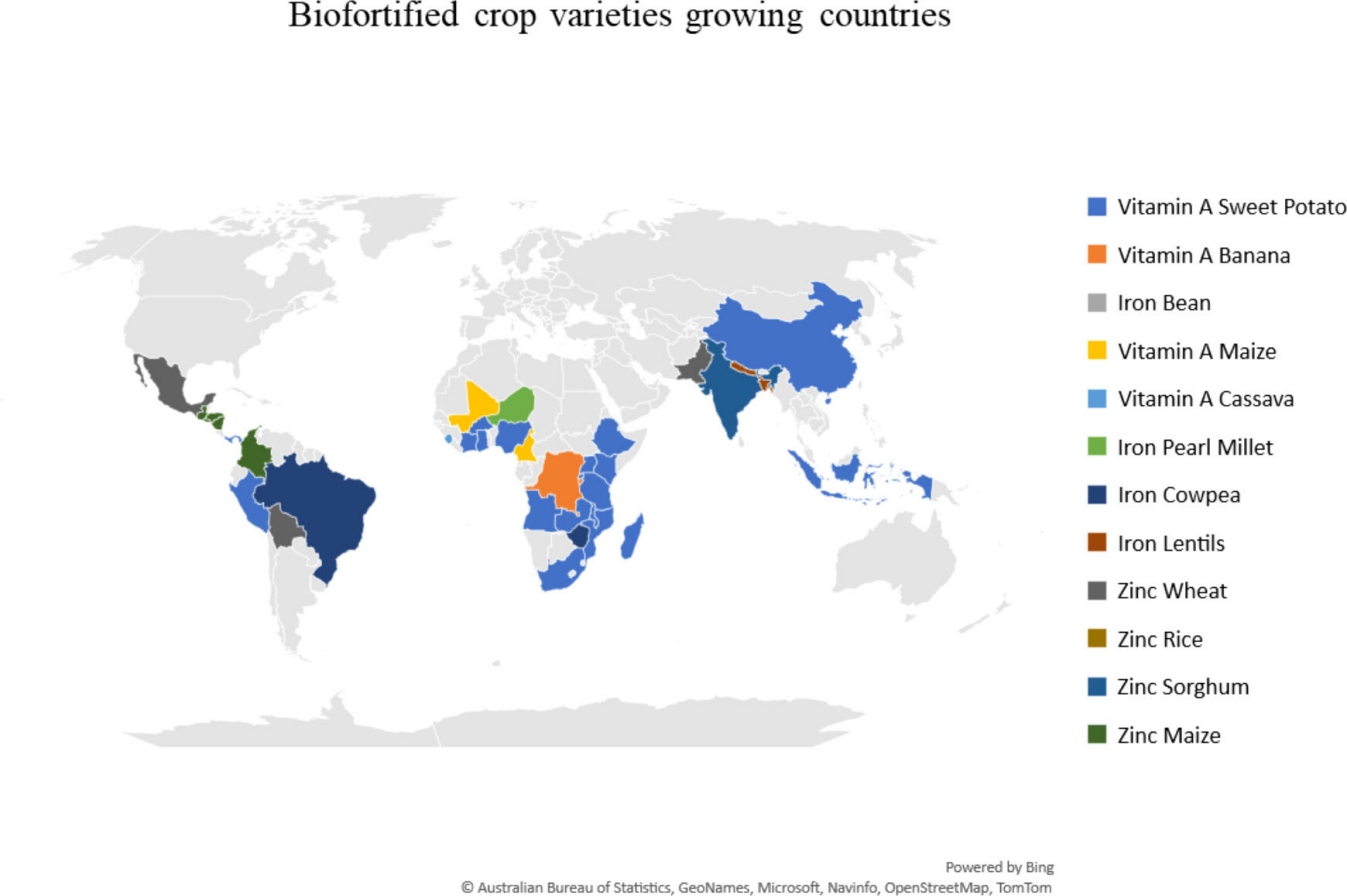
Biofortified crop cultivars grown in The Global South (source: CGIAR HarvestPlus).

The lack of precision phenotyping methods and accessibility for a variety of crops made the coordinated development of genomic markers for nutritional traits difficult. The preliminary list of usable DNA-validated markers is available elsewhere (https://excellenceinbreeding.org/toolbox/tools/kasp-low-density-genotyping-platform). This information supports the existing information about biofortified germplasm, varietal development, and releases (Andersson et al., 2017; Virk et al., 2021). Globally, more than 420 mineral- and vitamin-rich cultivars have been released to date (**Figure 2**). About 23 Zn-rich wheat cultivars were released in South Asia, with 2.2 million households currently growing these cultivars. Fifteen Zn rice cultivars are being cultivated by 2.4 million households across South Asia and Indonesia. For Zn maize, 11 cultivars are grown by 1 million in Colombia and Central America. Iron bean cultivars are the second highest in number (75) and reached about 3 million households in the sub-Saharan Africa and Latin America. About 10 iron pearl millet cultivars were released in Africa and India with 1 million households growing them nowadays. More than 20 provitamin A cassava cultivars were released and reached 2.6 million people in sub-Saharan Africa and Brazil. Sweet potato has the highest number of biofortified released cultivars in the Global South, reaching about 1.3 million people. The different cultivars reaching households may vary owing to its proportional farm adoption of nutrition-rich cultivars besides regular varieties and per-capita consumption in target populations (**Figure 3**).

## Bioavailability evidence for iron, zinc, and provitamin A

**Figure 4:**
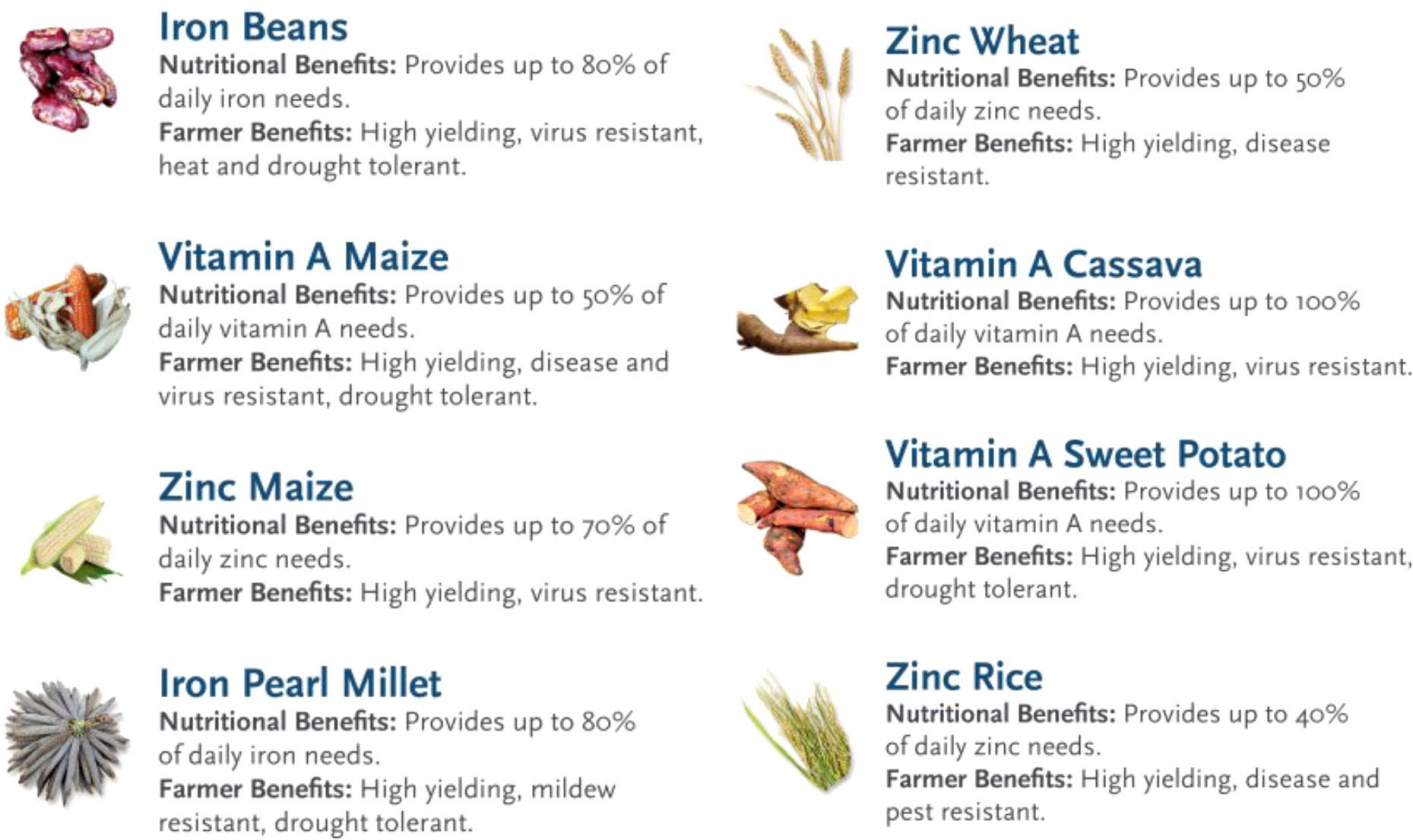
Detail of the nutrient-rich crops and their bioavailability potential and key farmer-preferred traits (source: CGIAR HarvestPlus).

Bioavailability is the portion of a nutrient used or stored by the human body after the target food’s digestion and absorption. This is a very complex trait to measure because many factors affect this process including diet matrix, individual food habits, and other communicable and non-communicable diseases that can play a key role in nutrient absorption. For instance, fat in the food or meal improves provitamin A carotenoid absorption, while disease inhibits its absorption. The degree of specific nutrient absorption can intensely vary depending on the type or form of nutrients. For example, there are two forms of iron in diets, i.e., heme (in animal-derived food) and non-heme (in plant-derived food). The last form of Fe was present in nutrient-rich crops and biofortified cultivars. Non-heme Fe in grains potentially brings other possible inhibitors such as phytic acid, tannins, and polyphenols, which are found in cereal and legumes. They can affect Fe absorption besides having other biological benefits (Dasa and Tilahun, 2018). Hence, regular consumption of biofortified cultivars on nutrition and health outcomes is required to systematically assess the impact of consuming nutrient-dense crops. In such an assessment, the nutritional benefits of the given nutrient-rich cultivars of a given crop are always compared with a regular (low nutrient) cultivar. Several independent bioavailability studies were conducted in bean, cassava, maize, pearl millet, rice, sweet potato, and wheat for Fe, Zn, and provitamin A (PVA). Rigorous bioavailability and efficacy research provides evidence that additional Fe, Zn, and PVA in biofortified crops brought public health benefits (Virk et al., 2021). **Figure 4** explains the potential target bioavailability in nutrient-rich crop cultivars to meet the recommended daily allowance (RDA) that has measurable human health and nutritional impacts. To date, about 15 bioavailability and efficacy randomized intervention trials were published (CAST, 2020). Briefly, Fe-rich pearl millet and bean cultivars contributed up to 80% of the daily requirement, while 40%, 50%, and 70% of daily Zn could be provided by Zn-rich maize, rice, and wheat, respectively. For PVA crops, up to 50% daily requirement could be met by consuming orange maize and 100% could be met from cassava and orange flesh sweet potato (Birol et al., 2021).

Consuming Fe-rich beans for at least 4 months significantly enhanced the Fe levels (hemoglobin and total body Fe) among adolescents in Rwanda (Haas et al., 2016). Similar Fe improvement was noticed among children in Mexico after 6 months of consuming Fe beans (Finkelstein et al., 2019a; Finkelstein et al., 2019b). In India, students (12–16 years old) with Fe deficiency at the beginning of the study fed with Fe-rich pearl millet diet significantly improved iron level after 4 months compared with control pearl millet diet; thus, feeding Fe-rich pearl millet improves iron level in school-aged children (Finkelstein et al., 2015). Fe-rich pearl millet and bean cultivars also enhanced physical and cognizant activities among fed students compared to other cultivars of the same crops (Murray-Kolb, 2013; Murray-Kolb et al., 2017; Scott et al., 2018). Elsewhere, Fe bioavailability was comprehensively reviewed (La Frano et al., 2013; La Frano et al., 2014; Boy et al., 2017). Zn absorption from flour derived from Zn-wheat cultivar was 80% of the control post-harvest fortified Zn flour among healthy adult women (Signorell et al., 2019). Another study indicated that consuming biofortified cultivar Zn led to a 25% increase compared to using post-harvest fortified wheat flour for Zn (CAST, 2020). Similarly, it was estimated that 8% to 20% of the PVA in PVA-rich crops (cassava, maize, and sweet potato) are bioavailable.

## Genetically modified Fe- and Zn-biofortified crops

High phytate content in the grains is a major barrier to Fe biofortification. Transgenic maize carrying endosperm-specific overexpression of soybean *ferritin* gene in the genetic background of low-phytate (*lpa1-1*) mutant accumulated up to 70 μg g^−1^ seed Fe and more than twofold improvement in bioavailable Fe. The level of bioavailable seed Fe greatly exceeded and closely approached values recommended to have a nutritional impact on target populations. Moreover, high-iron *lpa1-1* seeds relative to WT seeds showed a higher germination rate and seedling vigor, a useful genetic resource in maize breeding (Aluru et al., 2011). Low-phytate mutants, with some exceptions (Bregitzer and Raboy, 2006; Yuan et al., 2007; Campion et al., 2009), show lower seed germination and seedling vigor, and yield low compared to WT (Meis et al., 2003; Pilu et al., 2003; Oltmans et al., 2005; Shi et al., 2007).

Field evaluation of genetically modified (GM) rice event (NASFer-274) containing *OsNAS2* and *SferH-1* genes and evaluated in two countries showed 15 μg g^−1 ^Fe and 45.7 μg g^−1 ^Zn in polished grain, which exceeded HarvestPlus’ breeding target of 13 μg g^−1 ^Fe and 28 μg g^−1 ^Zn (Bouis et al., 2011), without any yield penalty or altered seed quality and trait remaining stable across genetic backgrounds (Trijatmiko et al., 2016). Fe and Zn content in polished T_1_ seeds from transgenic rice overexpressing *HvNAS1* increased by more than threefold and by twofold, respectively. Fe and Zn content also increased in both polished and brown T_2_ seeds (Masuda et al., 2009). Bioavailability of micronutrients is an issue for biofortification programs. Transgenic rice overexpressing *OsNAS1* fused to a glutelin promoter increased Fe in brown and milled rice seeds, with twice more bioavailable Fe than WT seeds (Zheng et al., 2010). Transgenic rice expressing soybean *ferritin* gene under the control of the glutelin promoter, relative to WT, has increased Fe and Zn content in the polished grain in an elite *indica* line (Vasconcelos et al., 2003).

## Genetically modified “provitamin A” biofortified crops

### Grain crops

Transgenic approaches have been used as tools to complement conventional breeding techniques to enhance the nutritional quality of food crops. Overexpression of *crtB* and *crtl* under the control of an endosperm-specific promoter increased total carotenoids by up to 34-fold with a substantial accumulation of β-carotene in the maize endosperm, and high β-carotene transgenic events were stable across generations (Aluru et al., 2008). Overexpression of *ibOr* gene in maize inbred lines (H145 and H95) under the control of a seed-specific promoter significantly increased total carotenoid and β-carotene. The total carotenoid and β-carotene content in H145-IbOr.10 increased up to 10.36- and 15.11-fold, respectively, compared to the WT (H145-WT), while in H95-IbOr.6 relative to WT (H95-WT), total carotenoids and β-carotene respectively increased by 5.58- and 7.63-fold (Tran et al., 2017). Simultaneous modification of three separate metabolic pathways resulted in transgenic maize’s 169-, 6-, and 2-fold increase in the amount of β-carotene, ascorbate, and folate, respectively, and the levels of engineered vitamins remained stable at least through to the T_3_ homozygous generation (Naqvi et al., 2009).

Rice seed, unlike maize, is devoid of β-carotene. Transgenic “Golden Rice” was bred by introducing *Zmpsy1* and *crtl* into Kaybonnet, an US temperate *japonica* rice (Ye et al., 2000; Paine et al., 2005). Thereafter, the “Golden Rice” (GR2E) characteristic was bred into Asian cultivars *via* marker-assisted backcross breeding (Mallikarjuna Swamy et al., 2021). The milled rice from GR2E contributed up to 89%–113% and 57%–99% of the estimated average requirement for vitamin A for preschool children in Bangladesh and the Philippines, respectively, according to efficacy trials (Mallikarjuna Swamy et al., 2019). A few Asian countries have already released Bt brinjal (eggplant, *Solanum melongena*) and GM maize for commercial production (Shelton et al., 2018; https://allianceforscience.cornell.edu/blog/2019/01/gmo-corn-transforming-farmers-lives-philippines/). The Philippine government has approved the release of provitamin A-rich (β-carotene) Golden Rice cultivar in their country (Steur et al., 2022; https://www.irri.org/news-and-events/news/philippines-becomes-first-country-approve-nutrient-enriched-golden-rice). “Golden Rice” has been judged as safe to eat by the US Food and Drug Administration (Owens, 2018).

Transgenic sorghum lines containing provitamin A genes (*PSY1* + *CRTI* genes, +/− *CRTB* gene) and vitamin E (*HGGT* gene) from different genetic backgrounds revealed significantly greater provitamin A content (5.9–28.6 mg g^−1^ FW) compared to their respective null segregants (0.4 –1.2 mg g^−1^ FW) or WT (0.73 mg g^−1^ FW). A general increase in vitamin E content, 14.9 –36.2 mg g^−1^ FW in transgenic versus 14.2–32.4 mg g^−1^ FW in null/WT, was also noted. Fe and Zn accumulation among provitamin A biofortified sorghum events varied from 27.65–63.59 mg g^−1^ FW to 28.44–48.85 mg g^−1^ FW, respectively, with the highest levels achieved in the sorghum event, Tx430. The corresponding increase in vitamin E levels improved provitamin A stability over storage (Debelo et al., 2020). An earlier report also revealed that the *HGGT* gene stacked with carotenoid biosynthesis genes mitigated oxidation of β-carotene during storage (Che et al., 2016). More recently, porridges made from biofortified sorghum grains containing multiple transgenes (*HGGT* to increase vitamin E accumulation and stabilize provitamin A carotenoids during storage, *CTR1* to increase provitamin A biosynthesis, *PSY1* or *CRTB* to increase flux through the carotenoid pathway, and *PhyA* to decrease phytate) revealed greater amounts of carotenoids compared to the corresponding null segregants and WT control. Steeping prior to porridge production to pre-activate phytase substantially reduces phytate content, alters the profile of inositol phosphate conversion products, reduces phytate:Fe and phytate:Zn molar ratios, and enhances mineral bioavailability. Micellarization efficiency and the bioaccessible fraction of provitamin A carotenoids were 2,300% greater in transgenic events compared to the corresponding null segregants and WT and provided 53.7% of a 4- to 8-year-old child’s vitamin A estimated average requirement in a standard 200-g serving of porridge. Thus, it is feasible to enhance micronutrient content and bioaccessibility by adapting a combination of strategies (Dzakovich et al., 2022).

Transgenic soybean lines contain the *β*-conglycinin promoter:*Phytoene synthase*-*2A*-*Carotene desaturase*/t35S gene cassette into the genome of soybean cultivar. “Kwangan” enhances β-carotene productions that ranged from 170.47 to 213.58 μg g^−1^ (Qin et al., 2017). An earlier report of transgenic soybean lines carrying two carotenoid biosynthetic genes, *Capsicum phytoene synthase* and *Pantoea carotene desaturase*, accumulated 146 µg g^−1^ of total carotenoids, ~ 62-fold higher than non-transgenic seeds, of which 112 µg g^−1^ was β-carotene (Kim et al., 2012).

### Root, tuber, and plantain crops

Provitamin A-biofortified transgenic Cavendish bananas containing *MtPsy2a* gene exceeded the target level of 20 μg g^−1^ with one line reaching 55 μg g^−1^ β-carotene equivalent fruit dry weight. The early activation of the rate-limiting enzyme in the carotenoid biosynthetic pathway and extended fruit maturation time are crucial factors to achieve optimal provitamin A concentrations in banana fruit (Paul et al., 2017). The project “Banana21” is currently underway to popularize Cavendish bananas in Uganda and surrounding countries through the generation of farmer- and consumer-acceptable edible bananas with significantly increased fruit levels of provitamin A (Paul et al., 2018).

BioCassava Plus (BC+) has developed transgenic cassava resistance to viral diseases, increased nutrients (Fe, Zn, vitamin A, protein) and shelf life, and reduced toxic cyanogenic glycosides to safe levels. Limited field trials in Puerto Rico and Nigeria and *ex ante* impact analyses demonstrate the efficacy of using transgenic technology for biofortification of cassava (Sayre et al., 2011). Roots harvested from transgenic cassava expressing *DXS* and *crtB* genes mediated by the patatin-type 1 promoter accumulated carotenoids up to ≤50 μg g^−1^ RDW, a 15- to 20-fold increase relative to non-transgenic cassava, ~85%–90% of which were β-carotene and display delayed onset of postharvest physiological deterioration. The inverse correlation between β-carotene and dry matter content, however, reduces 50%–60% dry matter in high carotenoid accumulating storage roots. This also resulted in reduced starch content but total fat, triacylglycerols, soluble sugars, and ascorbic acid levels increased, thus pinpointing the adverse effect of cassava biofortification on root dry weight and starch content (Beyene et al., 2018). Transgenic potato containing a bacterial mini-pathway (*CrtB*, *CrtI*, and *CrtY*) for β-carotene in a tuber-specific manner resulted in “golden” potato tubers that display a deep yellow to orange fleshed tuber phenotype containing β-carotene (> 3,000-fold over the WT), lutein (30-fold), β,β-xanthophylls (9-fold), and α-carotene (Diretto et al., 2007). Scaling up of provitamin A-rich banana, sweet potato, and cassava reached 8.5 million households in East Africa. More nutritious food systems supported by continued investment to enhance multiple nutrient targets into genetic background adapted to changing climates and increase investment in the inputs and marketing infrastructure are needed for popularization of nutrient-dense, vegetatively propagated crops (Low et al., 2022).

Overall, the proof of concept has demonstrated that it is achievable to develop GM crops with enhanced provitamin A. Equally important observations include the idea that seed chemistry was alike between GR2E and the near-isogenic control except for β-carotene and related carotenoids (Mallikarjuna Swamy et al., 2019), suggesting that golden rice cultivars are comparable to conventional rice for grain quality and nutritional traits and safe for human consumption. The drought-tolerant soybean (IND-ØØ41Ø-5) and wheat (IND-ØØ412-7) containing events *HaHB4* were compositionally equivalent to their non-transgenic parental controls (Ayala et al., 2019; Chiozza et al., 2020). Furthermore, a 42-day broiler feeding trial involving isoenergetic diets containing 40% flour from transgenic wheat and its non-GM counterpart “Cadenza” support the nutritional equivalence of transgenic lines when compared with conventional wheat. A few significant differences noted with respect to a commercial variety were however associated with the genetic background (Miranda et al., 2022). Antinutrients such as phytic acid reduce seed minerals including Fe and Zn bioavailability. Yet, phytase seems to have no direct influence in grain mineral bioavailability. No significant differences in seed chemistry were noted between transgenic wheat containing phytase gene and its non-transgenic counterpart. However, a significant increase in phytase activity as well as iron and zinc bioaccessibility correlated well with a significant decrease in phytate (Akhtar et al., 2020). Argentina, Australia, Brazil, Colombia, New Zealand, Nigeria, and United States have recommended drought-tolerant GM wheat for food and feed uses (https://www.isaaa.org/gmapprovaldatabase/event/default.asp?EventID=574&=HB4%20Wheat). It therefore opens the way for the development of seed mineral (Fe and Zn)-dense and provitamin A-rich food crops to benefit the world’s poorest people. Bioavailability of nutrients is no longer an issue with the use of transgenic technologies.

## Bioaccessability regulation and acceptability of genetically modified nutrient-dense crops

Genetic engineering and gene editing offer means to develop nutritious crops for healthy human diets. Examples include modifying fats in oilseeds and starch in roots and tubers; increasing micronutrients and carotenoids in cereals, roots, fruits, and oil crops, or minerals and antioxidants in some vegetables; and reducing glucosinates in *Brassica* species or flavonoids in vegetables (Winkler, 2011; Adenle et al., 2012; Hefferon, 2015; Hirschi, 2020). Although cultivars resulting from crossbreeding undergo a relatively simple regulation legal framework, the release of GM crops follow a lengthy and costly regulatory approval according to biosafety laws that align with the Cartagena Protocol of the Convention of Biological Diversity. The biosafety appraisals include the application of permits for field trials, the assessments of hazards for the environment (including biodiversity) and human/animal health (based on a nutritional study), and regulations for the marketing of GM crops and their derived food. As indicated by Turnbull et al. (2021), the laws should not be static and must adjust according to evolving science-led knowledge based on the years of cultivating GM crops elsewhere. Although omics technology could be incorporated into the strategy for evaluating GM biofortified crops, because it allows comparing it with a non-genetically engineered cultivar (Gould et al., 2022), the analyses should give priority to the nutrient composition of the edible parts. This product-based approach depends on characteristics that are measured by advances in molecular biology. For example, changes in DNA may be easily determined by comparing the sequences of a GM biofortified crop and the plant from which it derived, thus confirming that there are no unintended alterations. Omics technology may also be used for comparing the produce from a new GM biofortified crop with that of available cultivars grown by farmers. Nevertheless, any analysis should be relevant to the product safety rather than to changes in the RNA, proteins, or methylations that do not affect the phenotype.

Public controversy often slows the process of releasing GM biofortified crops (Baulcombe et al., 2014; Adeyeye and Idowu-Adebayo, 2019), mostly due to nonscience-based campaigns (often invoking the “precautionary principle) by those claiming unproven hazards to the environment and human health, e.g., GM mustard in India (Pulla, 2016; Jayaraman, 2017; Kumar, 2017) or “Golden Rice” (Mayer, 2005; Wu et al., 2021b). This calls for a more pragmatic view for this debate considering both the best available knowledge and the need to deliver zero hunger as stated in the UN agenda 2030 (Gbashi et al., 2021). Access to information and public participation will facilitate any citizen to exercise and protect their right to sufficient, affordable, safe, and nutritious food (Wolson, 2007). As noted by Goodman (2014), there is no science-based evidence that any of the approved GM crops poses any harm to humans or other animals, and they show consistency with the recommendations of the Codex Alimentarius. Moreover, the most appropriate approach for assessing GM crops for their use as food or feed source should be case-by-case (Nica-Badea and Balacescu, 2021). “Golden Rice” (GR2E event) provides an interesting case study for compositional analysis of its grains along with those from a non-GM near-isogenic line (Mallikarjuna Swamy et al., 2019). The results of this research demonstrated that their grains differ only in the levels of provitamin carotenoids (the target trait), while the other compositional parameters of GR2E were within the range noted in crossbred rice cultivars with a history of safe human consumption. Further molecular characterization and safety assessments provided substantiated evidence that provitamin A rice lacks any known hazards (Oliva et al., 2020); therefore, its grain is safe to use as food and as a potent cost-effective option for fighting malnutrition.

## Modeling and food-based methods for assessing bioavailability and bioaccessibility of nutrients

Bioavailability, which is accepted as an important factor for health status, refers to the absorption level of an ingested nutrient from a given source that animals use in their metabolism. The different chemical forms of macro- and micronutrients affect their bioavailability. Furthermore, nutrients often interact among themselves or with other components of the diet at the absorption site, thus changing their bioavailability, resulting in a nil effect when enhancers and inhibitors cancel each other. Yates (2001) indicated that new bioavailability factors are defined as more chemical complexes of nutrients and food components are released into the market.

There are guidance documents that describe how to obtain data regarding nutrient bioavailability from proposed new sources (ANS et al., 2018). Knowledge on the bioavailability of nutrients from whole foods helps design foods, meals, and diets, particularly for distinct dietary targets related to health outcomes (Melse-Boonstra, 2020). Research on bioavailability using humans or animals is costly and limited by the number of bioactive compounds in the food or feed source(s). *In vitro* digestion systems, modeling, and analytical methods may be used as alternatives, but each has its pros and cons (King et al., 2000; Wood and Tamura, 2001; Carbonell-Capella et al., 2014; Marze, 2017). Hence, as indicated by Wang et al. (2021), an ideal method for determining all micronutrients is not feasible but rather each method under appropriate procedure can be effective for minimizing the differences between simulated results and reality.

Bioaccessibility, which includes bioactivity, is another term used to find out the nutritional efficiency of food and food formulas aimed for the betterment of human health. It gives important information regarding the most appropriate dosage and source of food matrices that ensure nutritional efficacy (Fernández-García et al., 2009). Bioaccessibility refers to the fraction of a compound released from its matrix in the gastrointestinal tract and is therefore available for intestinal absorption, while bioactivity encompasses the events related to the transport of a bioactive compound until it reaches the target tissue, its interaction with other biomolecules, the metabolism it will go through, and the biomarker generation along with the ensuing physiologic response. *In vitro* approaches are used for early bioaccessibility screening and are complemented by *in vivo* research that remains as the main criterion for determining bioaccessibility of nutrients and bioactive compounds. Any health claims should therefore include measurements of bioavailability, bioaccessibility, and bioactivity to show their significance.

## Genetic and molecular basis of nutrient transport and absorption in human

Multiple pathways affect the gene regulation of metabolism. Goodman (2010) indicated that the enzymes digesting basic carbohydrates, proteins, and fats in the gastrointestinal tract are well-known. Such a knowledge provides insights into the digestion and absorption of nutrients. The metabolism of carotenoid was also elucidated and biochemically characterized in the 2000s (von Lintig, 2010). The molecular basis for retinol esterification enzymatic activity was further revealed recently through studying mutations in selective proteins that influence vitamin A in human patients (Chelstowska et al., 2016).

Genetic variation among human beings determines nutrient efficacy and tolerance or intolerance, as well as affects the nutrient intake (Stover, 2007). SNPs modulate the inter-individual variation for the bioavailability of vitamins A, D, and E; lutein; lycopene; and phytosterols (Borel and Desmarchelier, 2018). These SNPs in genes are related to the intestinal uptake and transport of vitamins, carotenoids, and phytochemicals in humans. Although each SNP effect may be low, a combination of them accounts significantly for interindividual variability. Such an example includes the 28 SNPs from 11 genes that are involved in the modulation of the vitamin E bioavailability inter-individual variation.

The nutrients and genomes often interact with the former controlling distinct nutrient utilization and the latter modifying genome expression, stability, and viability (Stover, 2007). While nutrients alter human phenotypes by inducing gene expression, human gene variants affect important metabolic pathways and the nutrient ability to interact with them (Paoloni-Giacobino et al., 2003). A better understanding of both provides knowledge about how metabolic homeostasis is kept and how to develop a diet therapy for human health. In this regard, nutrition genetics has two main fields of study, namely, nutrigenetics and nutrigenomics (Carrillo-Espel et al., 2016). Nutrigenetics studies the interaction between genes and dietary nutrients, while nutrigenomics investigates how food compounds regulate genes. Both contribute to personalized diets informed by molecular, genetic, and epigenetics features, and aim to prevent metabolic diseases by knowing the patient’s genome. As noted by Uthpala et al. (2020), innovative functional foods may be based on genetic patterns, thereby enhancing personalized diets for human consumption. Both nutrigenetics and nutrigenomics are still on the development stage, and their further advancement will facilitate eating foods that are beneficial to human DNA.

## Concluding remarks

Malnutrition, in all its forms, imposes a staggering cost to human welfare and the nation’s economy and is a major impediment to achieving Sustainable Development Goal 2 “Zero Hunger” by 2030. Crossbreeding and genetic engineering techniques have been successfully applied to develop nutrient-dense (Fe, Zn) and provitamin A-rich staple food crops. Fe- and Zn-rich beans, pearl millet, rice, wheat, cassava, and sweet potato cultivars, developed through cross breeding and selection, are grown and widely consumed in the Global South. Genetic engineering has been applied to develop provitamin A-rich “Golden Rice” and dessert bananas. The provitamin A trait used for developing “Golden Rice” has been successfully transferred into several locally adapted cultivars in Asia and released in the Philippines. Enhancing multiple nutrient targets through genetic engineering is now achievable as evidenced in maize grains rich in provitamin A, ascorbate, and folate and sorghum grains rich in both provitamin A and vitamin E.

Plants have developed tightly regulated mechanisms to achieve nutrient acquisition, transport, and storage in plant organs and relocation from source to sink organs at critical stages of plant growth and development. A comprehensive understanding of the cross-talk s between the signaling pathways integrating the homeostasis of essential mineral nutrients at the molecular level could allow researchers to develop a more efficient breeding strategy to enhance nutrient levels in plant edible parts.

Because of global warming, today’s food is less nutritious. Future research should focus on (i) identifying nutrient-dense germplasm, including sources from crop wild relatives, under elevated CO_2_, drought, and heat stress; (ii) assessing and identifying more efficient root microbiomes, rhizomes, and mycorrhiza fungi adapted to harsh environments; (iii) unlocking the complex connect between regulatory layers of homoeostasis of mineral nutrients balancing concentrations of essential micronutrients both at the cellular and systemic levels; (iv) understanding where and how micronutrients accumulate in seed crops, as well as managing variation for aleurone layers in germplasm; (v) identifying and exploiting genetic variability to reveal genomic regions controlling micronutrients that do not colocalize with increased nutrient absorption; (vi) integrating low-phytate germplasm with those high in essential micronutrients to develop biofortified cultivars; (vii) using genome-wide association genetics to reveal major QTLs, putative and functionally characterized genes, and associated SNPs to select for multiple elements with improved genetic background; (viii) identifying and exploiting positive pleiotropic loci impacting multiple target traits; (ix) unlocking genetic and molecular variation to reveal the inverse relationship between provitamin A, tuberous root dry weight, and starch content in root crops such as cassava; (x) enhancing the nutritional value of endosperm through biotechnological interventions to develop nutrient-dense grain or derived flour; (xi) using and promoting genetically engineered crops through multiple gene stacking for greater bioavailability and bioaccessibility of essential micronutrients; and (xii) unraveling the molecular basis of variation to avoid degradation of provitamin A during storage.

## Author contributions

SD: conceptualization, investigation, writing—original draft, and writing—review and editing. AG-O: investigation, writing—original draft, and editing. MG: investigation and writing—original draft. RO: conceptualization, project administration, investigation, writing—original draft, and writing—review and editing. All authors contributed to the article and approved the manuscript for submission.
